# Six new species of *Sporothrix* from hardwood trees in Poland

**DOI:** 10.3897/mycokeys.82.66603

**Published:** 2021-08-04

**Authors:** Agnieszka Ostafińska, Robert Jankowiak, Piotr Bilański, Halvor Solheim, Michael J. Wingfield

**Affiliations:** 1 Department of Forest Ecosystems Protection, University of Agriculture in Krakow, Al. 29 Listopada 46, 31-425 Krakow, Poland University of Agriculture Krakow Poland; 2 State Forets, Forest District Dynów, ul. Jaklów 2, 36-065 Dynów, Poland State Forets, Forest District Dynów Dynów Poland; 3 Norwegian Institute of Bioeconomy Research, P.O. Box 115, 1431 Ås, Norway Norwegian Institute of Bioeconomy Research Ås Norway; 4 Department of Biochemistry, Microbiology and Genetics, Forestry and Agricultural Biotechnology Institute (FABI), University of Pretoria, Pretoria 0002, South Africa University of Pretoria Pretoria South Africa

**Keywords:** 6 new species, bark beetle-associated fungi, *
Ophiostomatales
*, phylogeny, tree wounds

## Abstract

*Sporothrix* (*Sordariales*, *Ascomycota*) is a well-supported monophyletic lineage within the *Ophiostomatales*, species of which occur in a diverse range of habitats including on forest trees, in the soil, associated with bark beetles and mites as well as on the fruiting bodies of some *Basidiomycota*. Several species have also been reported as important human and animal pathogens. During surveys of insect- and wound-associated *Ophiostomatales* from hardwood trees in Poland, many isolates with affinity to *Sporothrix* were recovered. In the present study, six undescribed *Sporothrix* spp. collected during these surveys are characterized based on their morphological characteristics and multi-locus phylogenenetic inference. They are described as *Sporothrixcavum*, *Sporothrixcracoviensis*, *S.cryptarchum*, *S.fraxini*, *S.resoviensis*, and *S.undulata*. Two of the *Sporothrix* spp. reside in the *S.gossypina*-complex, while one forms part of the *S.stenoceras*-complex. One *Sporothrix* sp. is a member of lineage F, and two other species grouped outside any of the currently defined species complexes. All the newly described species were recovered from hardwood habitats in association with sub-cortical insects, wounds or woodpecker cavities. These species were morphologically similar, with predominantly asexual states having hyaline or lightly pigmented conidia, which produce holoblastically on denticulate conidiogenous cells. Five of the new taxa produce ascomata with necks terminating in long ostiolar hyphae and allantoid ascospores without sheaths. The results suggest that *Sporothrix* species are common members of the *Ophiostomatales* in hardwood ecosystems of Poland.

## Introduction

*Sporothrix* was established by Hektoen and Perkins (1900) based on the morphological description of the human pathogen, *Sporothrixschenckii*. Species of *Sporothrix* (*Ascomycota*, *Ophiostomatales*, *Ophiostomataceae*) were first accommodated in *Sporotrichum* (De Beurmann and Gougerot 1911). Until the latter half of the 20^th^ century, these fungi were also treated in various other genera, including *Cephalosporium*, *Cladosporium* (Hedgcock 1906; Münch 1907; Lagerberg et al. 1927; Melin and Nannfeldt 1934; Siemaszko 1939; Davidson 1942; Bakshi 1950; Mathiesen-Käärik 1953; Hunt 1956), *Cylindrocephalum*, *Hormodendron* (Robak 1932), *Hyalodendron* (Goidànich 1935; Georgescu et al. 1948), and *Rhinotrichum* (Georgescu et al. 1948; Sczerbin-Parfenenko 1953), in order to accommodate the asexual morphs of *Ophiostoma*. de Hoog (1974) published a monograph of the *Sporothrix* species and proposed the placement of *S.schenckii* as the asexual morph of *O.stenoceras*. That monograph expanded the concept of *Sporothrix* and included new *Sporothrix* species causing human infections as well as those associated with wood and bark beetles.

de Hoog et al. (1985) recognized that *Sporothrix* is not a homogenous group. As DNA sequencing technology was applied to resolve taxonomic relationships for fungi, evidence emerged that *S.schenckii* is phylogenetically related to species of *Ophiostoma* (Berbee and Taylor 1992; Hausner et al. 1993, 2000). In these studies, species producing only sporothrix-like asexual states were treated as members of the *S.schenckii*–*O.stenoceras* complex in *Ophiostomasensu lato* (De Beer et al. 2003; Villarreal et al. 2005; Roets et al. 2006; Zipfel et al. 2006; De Meyer et al. 2008; Linnakoski et al. 2010; Kamgan Nkuekam et al. 2012). The genus *Sporothrix* was recently redefined and emended based on the analysis of partial 18S and 28S rDNA sequences for species in the *Ophiostomatales* (De Beer et al. 2016). *Sporothrix* was consequently separated from species of *Ophiostoma* and various complexes were defined within *Sporothrix*. *Sporothrix* is now defined as one of nine relatively clearly defined genera in the *Ophiostomataceae* (De Beer and Wingfield 2013; De Beer et al. 2013a, 2013b, 2016).

As currently recognized, *Sporothrix* includes 56 species (De Beer et al. 2016; Ngubane et al. 2018; Wang et al. 2019; Musvuugwa et al. 2020), which are characterized by their dark brown to black, globose ascomata with elongated necks up to 1600 μm, occasionally terminating in an ostiole, often surrounded by ostiolar hyphae. Ascospores are usually curved and lunate to reniform, without a sheath (De Beer and Wingfield 2013). The asexual states have conidiophores that proliferate sympodially and produce hyaline or occasionally pigmented conidia on denticulate conidiogenous cells (De Beer and Wingfield 2013).

*Sporothrix* includes a large assemblage of species that are widely distributed across various climatic zones of the world (De Beer and Wingfield 2013; De Beer et al. 2016). Species also occupy a wide range of habitats. The greatest numbers of species are found on bark, in the infructescences of *Protea* spp. and on the wood of different forest trees (e.g., Roets et al. 2008, 2009, 2013; De Errasti et al. 2016). Other species have been described from soil, bark beetles, ambrosia beetles, mites, and from the fruiting bodies of basidiomycetes (e.g., Constantinescu and Ryman 1989; Marmolejo and Butin 1990; De Meyer et al. 2008; Roets et al. 2008; De Errasti et al. 2016). Several species are also well-known as human and animal pathogens (Travassos and Lloyd 1980; Summerbell et al. 1993; Barros et al. 2004; Lòpez-Romero et al. 2011; Zhang et al. 2015).

Jankowiak et al. (2019a) conducted the first extensive survey of fungal associates of hardwood-infesting bark and ambrosia beetles in Poland. In the same year, *Ophiostomatales* associated with wounds on hardwood trees were also studied in Poland (Jankowiak et al. 2019b). These studies reported several *Sporothrix* species, which were apparently new to science, but names were not provided for them. In addition, one unknown *Sporothrix* species was isolated from cavities of woodpeckers in Poland (Jankowiak et al. 2019c). In this study, morphological characters and DNA sequence data for the ITS region (ITS1–5.8S–ITS2) and three protein coding genes (β-tubulin, calmodulin, translation elongation factor 1-α) were analyzed to characterize six new species of *Sporothrix*. These were compared with closely related known species and formal descriptions have been provided for them.

## Materials and methods

### Fungal isolates

The collection details for the isolates included in the present study (Table 1) are provided in previous studies (Jankowiak et al. 2019a, 2019b, 2019c). The cultures are maintained in the culture collection of the Department of Forest Ecosystems Protection, University of Agriculture in Krakow, Poland, and in the culture collection of the Natural Resources Institute Finland (Luke), Helsinki, Finland. The ex-type isolates and representative isolates of the new species described were deposited in the culture collection (**CBS**) of the Westerdijk Fungal Biodiversity Institute, Utrecht, The Netherlands. Dried cultures were deposited as holotype specimens in the Mycological Herbarium (**O**), Natural History Museum, University of Oslo, Norway.

**Table 1.** Isolates used in the present study.

Fungal species	Previous identification^A^	Isolate no.			Source	Site	GenBank accessions^E^			
		CBS ^B^	O-F^C^	KFL=NRFI^D^			ITS1-5.8S-ITS2	βT	TEF 1-α	CAL
*Sporothrixcracoviensis* sp. nov	*Sporothrix* sp. 7	CBS 147940		KFL17FRJTD	Adult of *Trypodendrondomesticum* on *Fagussylvatica*	Krzeszowice	MH283148	MH283365	MH283500	MH283526
		CBS 147939		KFL2114bRJTD	Adult of *Trypodendrondomesticum* on *Fagussylvatica*	Krzeszowice	MH283149	MH283366	MH283501	MH283527
		CBS 147941	O-F-258629	KFL2514aRJTD^F^	Adult of *Trypodendrondomesticum* on *Fagussylvatica*	Krzeszowice	**MW768963**	MH283367	MH283502	MH283528
		CBS 147942^F,T^	O-F-258628	KFL2514bRJTD	Adult of *Trypodendrondomesticum* on *Fagussylvatica*	Krzeszowice	**MW768964**	MH283368	MH283503	MH283529
*Sporothrixfraxini* sp. nov	*Sporothrix* sp. 8	CBS 147936^F,T^	O-F-258630	KFL21BS16bRJHV	Gallery of *Hylesinusvarius* on *Fraxinusexcelsior*	Zbylitowska Góra	MH283150	MH283370	MH283504	MH283530
		CBS 147938^F^	O-F-258631	KFL21BS16dRJHV	Gallery of *Hylesinusvarius* on *Fraxinusexcelsior*	Zbylitowska Góra	**MW768968**	MH283371	**MW768973**	MH283531
		CBS 147937		KFL21BS16cRJHV	Gallery of *Hylesinusvarius* on *Fraxinusexcelsior*	Zbylitowska Góra	MH283151	MH283372	MH283505	MH283532
*Sporothrixresoviensis* sp. nov	*Sporothrix* sp. 10	CBS 147927^F,T^	O-F-258632	KFL204ABRZN16AO	Wound on *Betulapendula*	Borownica	MH740962	MH741100	MH741189	MH741228
*Sporothrixcryptarchum* sp. nov.	*Sporothrix* sp. 11			KFL1097NOL16RJ	Wound on *Alnusincana*	Wierzchosławice	MH740963	MH741101	MH741190	MH741229
				KFL1146NDB16RJ	Wound on *Quercusrobur*	Ispina	MH740964	MH741102	MH741191	MH741230
		CBS 147935		KFL48716NDBRJ	Wound on *Quercusrobur*	Wierzchosławice	**MW768967**	MH741103	MH741192	**MW768977**
		CBS 147934^F,T^	O-F-258633	KFL410DB16bRJCU	Adult of *Cryptarchaundata*	Wierzchosławice	**MW768966**	MH741104	MH741193	MH741231
		CBS 147933^E^	O-F-258634	KFL404DB16aRJCU	Adult of *Cryptarchaundata*	Wierzchosławice	**MW768965**	MH741105	MH741194	MH741232
*Sporothrixundulata* sp. nov.	*Sporothrix* sp. 12	CBS 147931^E^	O-F-258636	KFL13NDB15bRJ	Wound on *Quercusrobur*	Wierzchosławice	MH740965	MH741106	**MW768974**	**MW768978**
		CBS 147930		KFL12NDBCZ15RJ	Wound on *Quercusrubra*	Wierzchosławice	MH740967	MH741108	MH741196	**MW768979**
		CBS 147928		KFL221NBK16RJ	Wound on *Fagussylvatica*	Czajowice	MH740970	MH741112	MH741199	MH741235
		CBS 147932		KFL430NDB16RJ	Wound on *Quercusrobur*	Ispina	MH740971	MH741113	MH741200	MH741236
				KFL1099NOLCZ16RJ	Wound on *Alnusincana*	Wierzchosławice	MH740973	MH741115	MH741202	MH741237
				KFL1140NDB16bRJ	Wound on *Quercusrobur*	Ispina	MH740975	MH741117	MH741203	MH741238
				KFL6117NWB17RJ	Wound on *Salixfragilis*	Babimost	**MW768970**	MH741119	MH741204	**MW768980**
		CBS 147929^F,T^	O-F-258635	KFL398DB16RJEG	Adult of *Epuraeaguttata*	Wierzchosławice	MH740976	MH741121	MH741205	MH741239
				KFL404DB16bRJCU	Adult of *Cryptarchaundata*	Wierzchosławice	**MW768969**	MH741124	MH741208	MH741242
*Sporothrixcavum* sp. nov	*Sporothrix* sp. 18	CBS 147943^F,T^	O-F-258637	KFL42215aDRJ	Cavity of *Dendrocoposmajor* on *Salixfragilis*	Kraków	MF782813	MF782850	**MW768972**	**MW768976**
			O-F-258638	KFL35614DRJ^F^	Cavity of *Dendrocoposmedius* on *Malusdomestica*	Książ Wielki	MF782814	MF782851	**MW768971**	**MW768975**

^A^ Isolates collected and identified during previous surveys in Poland (Jankowiak et al. 2019a, 2019b, 2019c). *Sporothrix* sp. 18 in the study of Jankowiak et al. (2019c) was labelled as *Sporothrix* sp. ^B^CBS Westerdijk Fungal Biodiversity Institute, Utrecht, The Netherlands. ^C^ Herbarium of the Natural History Museum, University of Oslo, Norway, ^D^ KFL Culture collection of the Department of Forest Ecosystems Protection, University of Agriculture in Krakow, Poland; NRIF The Natural Resources Institute Finland (Luke), Helsinki, Finland. ^E^ ITS1-5.8S-ITS2-ITS2 = the internal transcribed spacer 1 and 2 regions of the nuclear ribosomal DNA gene, 5.8S rRNA gene; βT= Beta-tubulin; TEF1-α = Translation elongation factor 1-alpha; CAL = Calmodulin. ^F^ Isolates used in growth and morphological studies; ^T^type strain Sequences obtained during the survey in this study are indicated in bold.

### Microscopy and growth studies

Morphological characters were examined for selected isolates as well as for the herbarium specimens selected as types. Cultures were grown on 2% Malt Extrat Agar (**MEA**) made up of 20 g Bacto malt extract, 20 g agar Bacto agar powder (Becton Dickinson and Company, Franklin Lakes, USA) in 1 l deionized water. In attempts to induce the formation of ascomata, autoclaved twigs of host trees including the bark were placed at the centres of agar plates containing MEA. Fungal cultures were derived from single spores. To promote the production of ascomata, single conidial isolates were crossed in all possible combinations, following the technique described by Grobbelaar et al. (2009). These cultures were incubated at 25 °C and monitored regularly for the appearance of fruiting structures.

Morphological features were examined by mounting fungal tissue in 80% lactic acid on glass slides, and fruiting structures were observed using a Nikon Eclipse 50*i* microscope (Nikon Corporation, Tokyo, Japan) with an Invenio 5S digital camera (DeltaPix, Maalov, Denmark) to capture photographic images. Microscopy followed the technique described by Kamgan Nkuekam et al. (2011). Colour designations were based on the colour charts of Kornerup and Wanscher (1978).

For each taxonomically relevant structure, fifty measurements were made, when possible, using the Coolview 1.6.0 software (Precoptic, Warsaw, Poland). Averages, ranges and standard deviations were calculated for the measurements, and these are presented in the format ‘(min–)(mean–SD)–(mean+SD)(–max)’.

Growth characteristics for the novel species were determined by analysing the radial growth for 12 isolates (two for each species) (Table 1). Agar disks (5 mm diam.) were cut from the actively growing margins of fungal colonies and these disks were placed at the centres of plates containing 2% MEA. Four replicate plates for each of the six putative new species were incubated at temperatures between 5, and 35 °C at 5 °C intervals. The radial growth (two measurements perpendicular to each other per plate) was determined 14 d after inoculation, and growth rates were calculated as mm/d.

### PCR, sequencing and phylogenetic analyses

**Figure 1.**
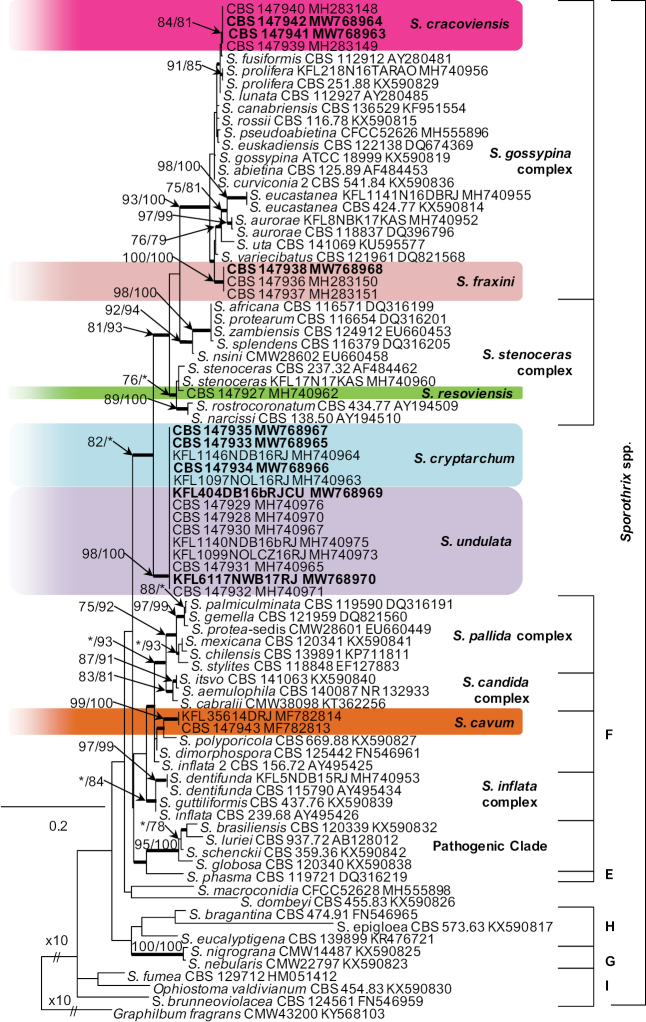
Phylogram obtained from Maximum Likelihood (ML) analyses of the ITS1-5.8S-ITS2 data for the *Sporothrix* spp. Sequences obtained during this study are presented in bold type. The Bootstrap values ≥ 75% for ML and Maximum Parsimony (MP) analyses are presented at nodes as follows: ML/MP. Bold branches indicate posterior probabilities values ≥ 0.95 obtained from Bayesian Inference (BI) analyses. * Bootstrap values <75%. The tree is drawn to scale (see bar) with branch length measured in the number of substitutions per site. *Graphilbumfragrans* represent the outgroup.

**Figure 2.**
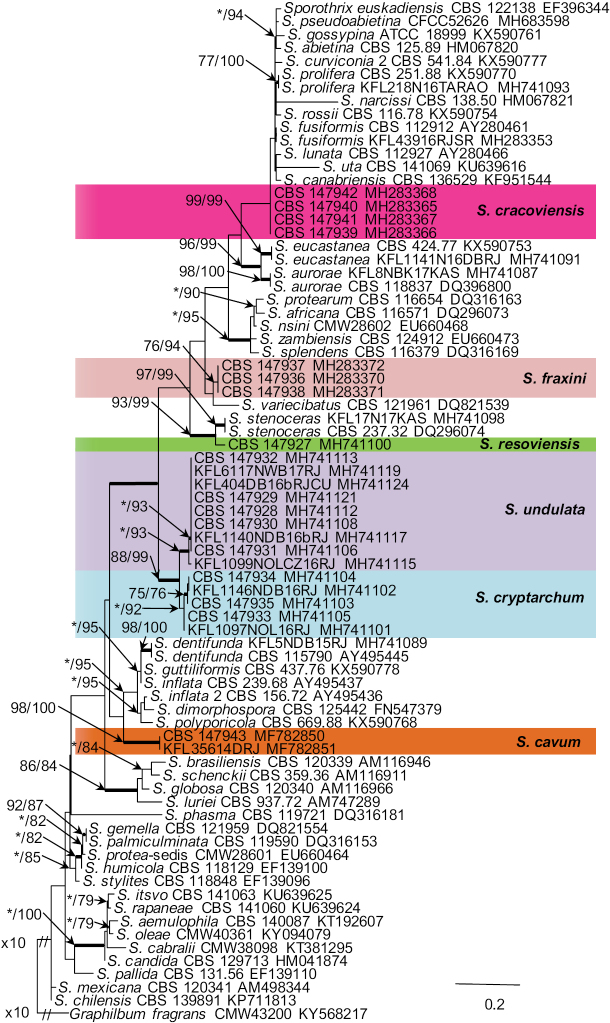
Phylogram obtained from Maximum Likelihood (ML) analyses of βT data for the *Sporothrix* spp. Sequences obtained during this study are presented in bold type. The Bootstrap values ≥ 75% for ML and Maximum Parsimony (MP) analyses are presented at nodes as follows: ML/MP. Bold branches indicate posterior probabilities values ≥ 0.95 obtained from Bayesian Inference (BI) analyses. * Bootstrap values <75%. The tree is drawn to scale (see bar) with branch length measured in the number of substitutions per site. *Graphilbumfragrans* represent the outgroup.

**Figure 3.**
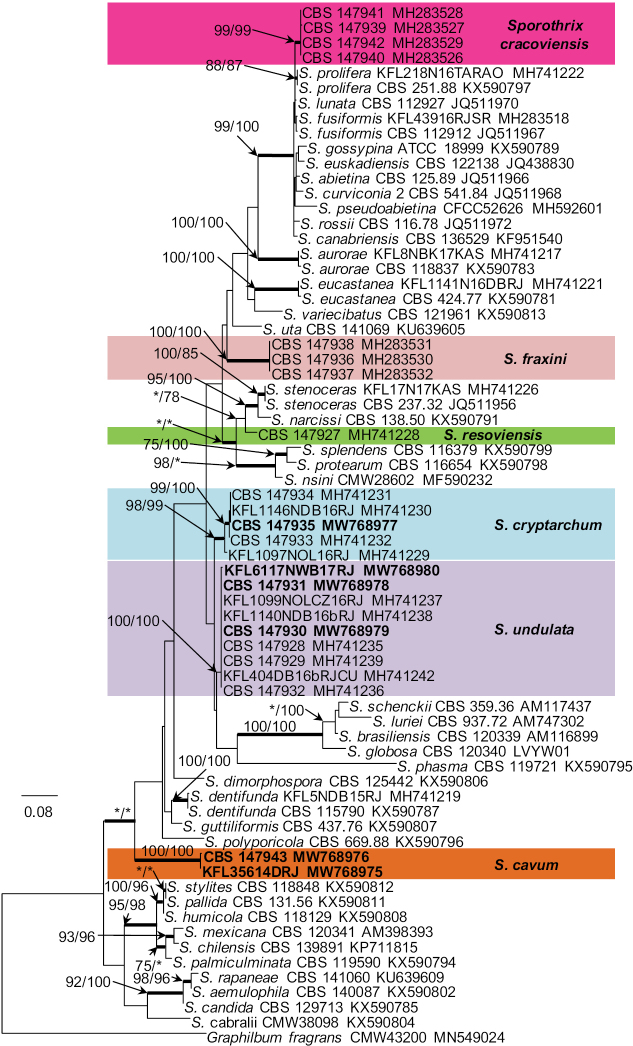
Phylogram obtained from Maximum Likelihood (ML) analyses of CAL data for the *Sporothrix* spp. Sequences obtained during this study are presented in bold type. The Bootstrap values ≥ 75% for ML and Maximum Parsimony (MP) analyses are presented at nodes as follows: ML/MP. Bold branches indicate posterior probabilities values ≥ 0.95 obtained from Bayesian Inference (BI) analyses. * Bootstrap values <75%. The tree is drawn to scale (see bar) with branch length measured in the number of substitutions per site. *Graphilbumfragrans* represent the outgroup.

**Figure 4.**
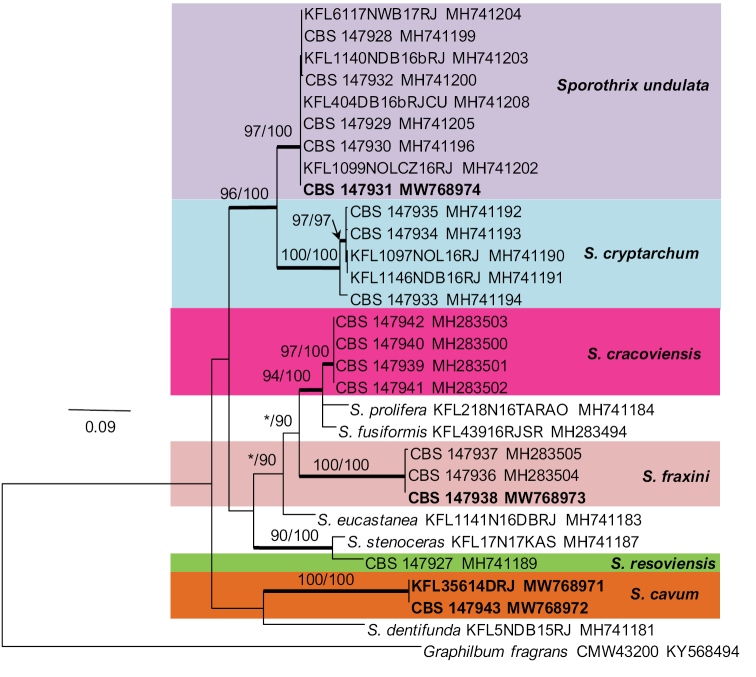
Phylogram obtained from Maximum Likelihood (ML) analyses of TEF1-α data for the *Sporothrix* spp. Sequences obtained during this study are presented in bold type. The Bootstrap values ≥ 75% for ML and Maximum Parsimony (MP) analyses are presented at nodes as follows: ML/MP. Bold branches indicate posterior probabilities values ≥ 0.95 obtained from Bayesian Inference (BI) analyses. * Bootstrap values <75%. The tree is drawn to scale (see bar) with branch length measured in the number of substitutions per site. *Graphilbumfragrans* represent the outgroup.

**Figure 5.**
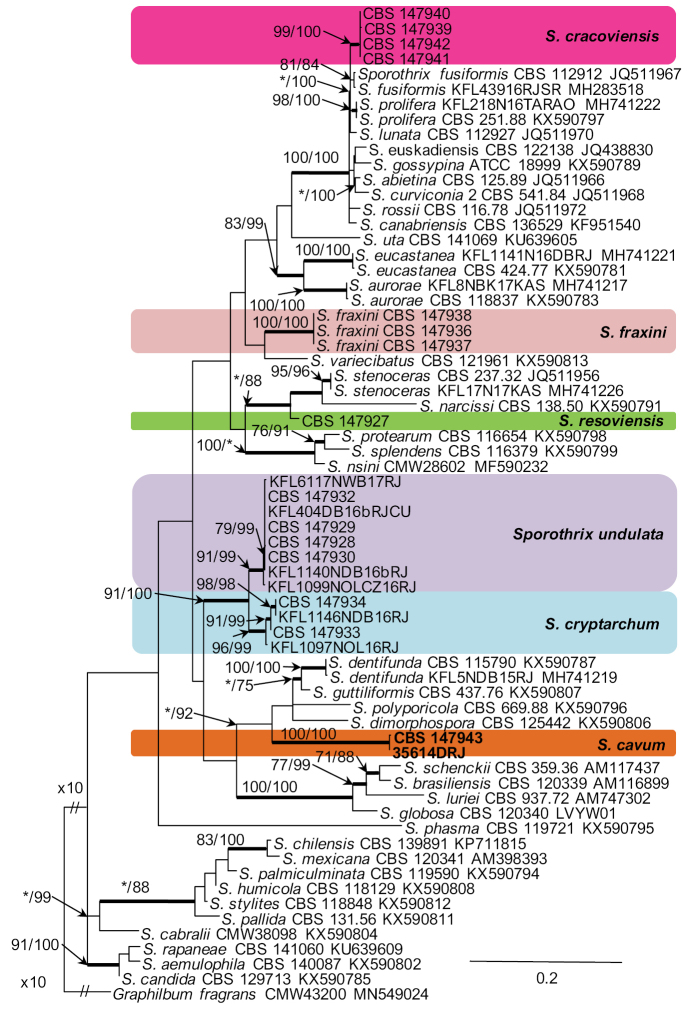
Phylogram obtained from Maximum Likelihood (ML) analyses of the combined βT and CAL sequences of the *Sporothrix* spp. Sequences obtained during this study are presented in bold type. The Bootstrap values ≥ 75% for ML and Maximum Parsimony (MP) analyses are presented at nodes as follows: ML/MP. Bold branches indicate posterior probabilities values ≥ 0.95 obtained from Bayesian Inference (BI) analyses. * Bootstrap values <75%. The tree is drawn to scale (see bar) with branch length measured in the number of substitutions per site. *Graphilbumfragrans* represent the outgroup.

DNA extractions were performed as described by Jankowiak et al. (2019d). For sequencing and phylogenetic analyses, four loci were amplified: the internal transcribed spacer region (ITS, consisting of ITS1, 5.8S, and ITS2), beta tubulin (βT), calmodulin (CAL), and the translation elongation factor 1-alpha (TEF1-α). The primers used for PCR and sequencing of the various gene regions were as follows: ITS1-F (Gardes and Bruns 1993) and ITS4 (White et al. 1990) for ITS; T10 (O’Donnell and Cigelnik 1997) or Bt2a together with Bt2b (Glass and Donaldson 1995) for βT; F-728F (Carbone and Kohn 1999) and EF2 (O’Donnell et al. 1998) were used for TEF1-α; CL1 and CL2a (O’Donnell et al. 2000) or CL3F and CL3R (De Beer et al. 2016) were used for CAL. PCR and sequencing protocols were as described by Jankowiak et al. (2019d), other than the annealing temperature being optimised for some individual reactions. All analyses were run independently for each gene partition (Figs 1–4). Resulting trees were visually compared for topological incongruence. Gene partitions showing no topological incongruence (βT, CAL) were combined and presented as a concatenated construct (Fig. 5).

For phylogenetic analyses, sequence alignments were performed using the online version of MAFFT v7 (Katoh and Standley 2013). The ITS, βT, CAL, and TEF1-α datasets were aligned using the E-INS-i strategy with a 200PAM/κ=2 scoring matrix, a gap opening penalty of 1.53 and an offset value of 0.00. The alignments were checked manually with BioEdit v.2.7.5 (Hall 1999). The resulting alignments and trees were deposited into TreeBASE (http://purl.org/phylo/treebase/phylows/study/TB2:S27966).

Phylogenetic trees were inferred for each of the datasets using three different methods: Maximum likelihood (ML), Maximum Parsimony (MP) and Bayesian inference (BI). For ML and BI analyses, the best-fit substitution models for each aligned dataset were established using the corrected Akaike Information Criterion (AICc) in jModelTest 2.1.10 (Guindon and Gascuel 2003; Darriba et al. 2012). ML analyses were carried out with PhyML 3.0 (Guindon et al. 2010), utilizing the Montpelier online server (http://www.atgc-montpellier.fr/phyml/). The ML analysis included bootstrap analysis (1000 bootstrap pseudoreplicates) in order to assess node support values and the overall reliability of the tree topology. The best evolutionary substitution model was GTR+I+G for ITS (-lnL = 4497.47), GTR+G for CAL (-lnL = 4112.25) and TEF1-α (-lnL = 4218.36), HKY+G for βT (-lnL = 2641.05) and HKY+I+G for combined βT-CAL (-lnL 6798.48).

MP analyses were performed using PAUP* 4.0b10 (Swofford 2003). Gaps were treated as fifth state. Bootstrap analysis (1000 bootstrap replicates) was conducted to determine the levels of confidence for the nodes within the inferred tree topologies. Tree bisection and reconnection (TBR) was selected as the branch swapping option. The tree length (TL), Consistency Index (CI), Retention Index (RI), Homoplasy Index (HI) and Rescaled Consistency Index (RC) were recorded for each analysed dataset after the trees were generated.

BI analyses using Markov Chain Monte Carlo (MCMC) methods were carried out with MrBayes v3.1.2 (Ronquist and Huelsenbeck 2003). Four MCMC chains were run for 10 million generations applying the best-fit model for each dataset. Trees were sampled every 100 generations, resulting in 100,000 trees. Tracer v1.4.1 (Rambaut and Drummond 2007) was utilized to determine the burn-in value for each dataset. The remaining trees were utilised to generate a 50% majority rule consensus tree, which allowed for calculating posterior probability values for the nodes.

## Results

### Phylogenetic Analyses

Alignments for the ITS dataset contained 575 characters; for the βT 303 characters; for CAL 543 characters; and for TEF1-α 812 characters; for the concatenated combined dataset 826 (including gaps), of which respectively 202, 123, 271, 439, 390 were parsimony-informative. The exon/intron arrangement of the βT data included exons 5 and 6, interrupted by intron 5. The exon/intron arrangement of the CAL data included exons 4 and 5, interrupted by intron 4. The aligned TEF1-α gene region consisted of intron 3 and exons 4 and 5, but lacked intron 4.

DNA sequence data were generated for 24 isolates considered in this study (Table 1). Blast analyses of the ribosomal DNA sequences placed all the isolates in *Sporothrix*. Based on phylogenetic analyses of the ITS (Fig. 1), the isolates emerged as six undescribed taxa. Phylogenetic analysis of the ITS indicated that the unknown species resided in two previously defined *Sporothrix* species complexes, including the *S.gossypina*- and *S.stenoceras*- species complexes, and lineage “F”. Additionally, isolates representing two new species grouped outside any of the currently defined species complexes (Fig. 1). Based on the availability of sequence data for these complexes, different datasets were assembled and analysed separately for each species complex.

Seven isolates from hardwood-infesting bark beetles identified as *Sporothrix* 7 and *Sporothrix* 8 by Jankowiak et al. (2019a) resided in the *S.gossypina*-complex (Fig. 1). All three gene regions (ITS, βt, CAL) separated *Sporothrix* sp. 8 from the other known species with strong statistical support (Figs 2–4). The ITS and βt gene regions grouped isolates of this species together with the ex- type isolate of *S.variecibatus*, while CAL gene region placed it with *S.aurorae* (Figs 1–3). Isolates representing *Sporothrix* sp. 7 had ITS sequences that were almost identical to the ITS sequences for *S.fusiformis*, *S.lunata* and *S.prolifera* (Fig. 1). In the βt and CAL trees (Figs 2, 3), *Sporothrix* sp. 7 formed lineages that clearly separated this species from the known species in the *S.gossypina* complex, and although there were differences in the βt sequence compared to other species, the node lacked statistical support (Fig. 2). The combined analyses of the βt and CAL datasets clearly distinguish *Sporothrix* sp. 7 and *Sporothrix* sp. 8 into separate lineages within the *S.gossypina*-complex (Fig. 5).

The single isolate from a wound on *Betulapendula* identified as *Sporothrix* sp. 10 by Jankowiak et al. (2019b), resided in *S.stenoceras*-complex and grouped closely with *S.stenoceras* sensu stricto based on analysis of ITS, βt, CAL, and TEF1-α gene regions (Figs 1–4). All three gene regions separated *Sporothrix* sp. 10 from *S.stenoceras*, although this separation was not statistically supported by the ITS gene region (Figs 1–4). The combined analyses of the βt and CAL datasets clearly distinguish *Sporothrix* sp. 10 into separate lineages within the *S.stenoceras*-complex (Fig. 5).

Two isolates from woodpecker cavities identified as *Sporothrix* sp. 18 by Jankowiak et al. (2019c), belonged to the lineage F defined by De Beer et al. (2016) based on the ITS tree. All the three gene regions (ITS, βt, CAL) separated *Sporothrix* sp. 18 from the other known species in lineage F with strong statistical support (Figs 1–4). The combined analyses of the βt and CAL datasets clearly distinguish *Sporothrix* sp. 18 into separate lineages within the *Sporothrix* spp. (Fig. 5).

Fourteen isolates from wounds on different species of hardwood trees and nitidulid beetles identified as *Sporothrix* sp. 11 and *Sporothrix* sp. 12 by Jankowiak et al. (2019b) did not group in any of the defined *Sprothrix* species complexes based on analysis of ITS gene region and formed a monophyletic lineage within *Sporothrix* (Fig. 1). Isolates of *Sporothrix* sp. 11 had ITS sequences that were identical with ITS sequences noted in *Sporothrix* sp. 12. In the βt, CAL, and TEF1-α trees (Figs 2–4), *Sporothrix* sp. 11 and *Sporothrix* sp. 12 formed well-supported lineages that clearly separated these two putative new species from each other. The combined analyses of the βt and CAL datasets also separated *Sporothrix* sp. 11 and Sporothrix sp. 12 from the other known species in *Sporothrix* spp. and also from each other (Fig. 5).

### Morphological characteristics

The six new taxa in *Sporothrix* emerging from the phylogenetic studies showed differences in colony colour. The cultures of *Sporothrix* spp. 7, 8, 10 and 11 were white. The cultures of *Sporothrix* sp. 12 were white or pigmented (white grey) whereas cultures of *Sporothrix* sp. 18 were greyish green. With the exception of *Sporothrix* sp. 7 cultures that had an optimum growth at 25 °C followed by 20 °C, all of the undescribed taxa displayed optimum growth at 25 °C followed by 30 °C.

All the new taxa emerging from this study produced micronematous conidiophores and hyaline or pigmented conidia formed holoblastically on denticulate conidiogenous cells. *Sporothrix* sp. 11 and *Sporothrix* sp. 12 were characterized by the formation of hyaline and pigmented conidia. Other than *Sporothrix* sp. 18, which remained asexual, a sexual morph was induced in all five of the other emerging taxa. Ascomata were black and globose with straight necks and up to 700 μm long. Ostiolar hyphae were well-developed and up to 74 μm long. Ascospores were allantoid (*Sporothrix* sp. 7, 8) or kidney-shaped (*Sporothrix* spp. 10–12), and they lacked sheaths.

## Taxonomy

### Sporothrix cracoviensis

Taxon classificationFungiOphiostomatalesOphiostomataceae

R. Jankowiak sp. nov.

CC070A7F-6EFE-555A-88B7-E6D1B556E89A

840460

Fig. 6

#### Etymology.

From Latin, referring to the capital of Małopolskie Voivodeship and the former capital of Poland (Cracovia in Latin, Kraków in Polish); the region where this fungus was collected.

#### Type.

Poland, Małopolskie Province, Krzeszowice, from adult *Tryopodendrondomesticum* beetle on *Fagussylvaticum*, January 2014, *R. Jankowiak* (O-F-258628 ***holotype***, culture ex-type CBS 147942).

#### Description.

Sexual and asexual structures produced on sterilised beech twigs on surface of malt agar in Petri dishes. *Ascomata* abundant, superficially or partly embedded in the agar, single or in groups; *ascomatal bases* black, globose, (66–)89–153(–245) μm diam., with brown hyphal hairs, 12 to 165 μm long and 1 to 1.8 μm wide at the base; *ascomatal necks* black, straight or curved, (187–)272–462(–611) μm long, diameter (9–)10.4–16.7(22.5) μm at the apex and (26.8–)29.9–50.5(–63.9) μm at the base. *Ostiolar hyphae* present, pale brown, septate, straight or slightly waved, tapering towards the apex or sporadically dichotomous branching at the tip, (7–)8–16(–22) in number (17.8–)29.6–48.4(–64.5) μm long, (0.3–)0.5–1(–1.5) μm at the apex and (1.2–)1.6–2.3–(3) μm at the base. *Asci* evanescent. *Ascospores* one-celled, allantoid in side view (2.8–)3.1–3.8(–5.1) × (1–)1.1–1.4(–1.6) μm, elliptical in front view (2.8–)3.1–4.2(–4.8) × (1–)1.2–1.5(–1.8) μm, sometimes with residual sheath up to 1 μm thick, accumulated in creamy-colored mass at the tip of the neck. *Conidiophores* hyaline, micronematous, simple or branched, straight, simple or branched, bearing several conidiogenous cells, either borne on vegetative hyphae or on upright hyphae. *Conidiogenous cells* blastics, cylindrical, terminal, lateral or intercalary, straight or curved, tapering towards the apex, swollen apical part forming conidia by sympodial proliferation on visible denticles, (4.2–)17.5–43.1(–72.2) μm long, (0.8–)1.1–1.7(–2.1) μm wide at the base. Apical part with denticles (0.8–)1.3–3.7(–7.3) μm long and (1.2–)1.7–3.7(–7.3) μm wide. Conidia hyaline, unicellular, smooth, obovoid to clavate, sometimes slightly curved, with slightly pointed bases, (2.8–)3.2–6.4(–8.7) × (1.1–)1.4–2.1(–2.7) μm, formed directly on denticles. *Culture characteristics*: Cultures showing optimal growth at 25 °C (1 mm/d) with somewhat slower growth by at 20 °C (0.8 mm/d), white, flat, floccose, growing in a circular pattern with smooth margins.

**Figure 6.**
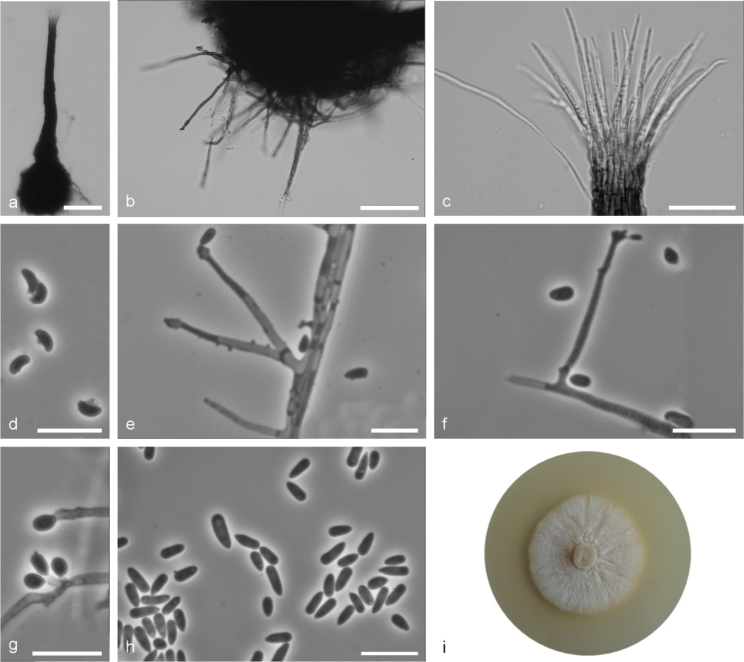
*Sporothrixcracoviensis* sp. nov. (CBS 147942) **a** ascoma **b** ascomatal base **c** ostiolar hyphae **d** ascospores **e, f** conidiogenous cell with an inflated cluster of denticles at the apex **g** conidiogenous cells arising directly from hyphae **h** conidia **i** fourteen-day-old culture on MEA. Scale bars: 50 μm (**a, b**), 25 μm (**c**), 10 μm (**d–h**).

#### Host tree.

*Fagussylvatica*.

#### Insect vector.

*Trypodendrondomesticum*, *T.signatum*.

#### Distribution.

Poland

#### Additional specimen examined.

Poland, Małopolskie Province, Krzeszowice, from adult *Tryopodendrondomesticum* beetle on *Fagussylvaticum*, January 2014, *R. Jankowiak* (O-F-258629, cultures CBS 147941).

#### Notes.

*Sporothrixcracoviensis* is phylogenetically distinct from the other *Sporothrix* species based on the βT, CAL and TEF1-α sequences. This species is closely related to *S.fusiformis*, *S.lunata* and *S.prolifera*. *Sporothrixcracoviensis* has smaller ascomatal necks (187–611 μm) compared to *S.fusiformis* (301–1168) μm (Aghayeva et al. 2004). Their conidial dimensions and shapes showed also differences. *Sporothrixfusiforme* has fusiforme conidia (Aghayeva et al. 2004), whereas *S.cracoviensis* has obovoid to clavate conidia. *Sporothixlunata* has also different shape of conidia (crescent) (Aghayeva et al. 2004) compared to *S.cracoviensis* (obovoid to clavate). In addition, *S.lunata* has smaller conidia (2.3–6.2 × 0.8–1.6 μm) (Aghayeva et al. 2004) compared to *S.cracoviensis* (2.8–8.7 μm × 1.1–2.7 μm). *Sporothrixprolifera* could be distinguished from *S.cracoviensis* by its smaller ascomatal base (*S.prolifera*: 65–90 μm (Kowalski and Butin 1989); *S.cracoviensis*: 66–245 μm) and smaller ascomatal necks (*S.prolifera*: 75–160 μm (Kowalski and Butin 1989); *S.cracoviensis*: 187–611 μm). In addition, *S.prolifera* has shorter ostiolar hyphae (*S.prolifera*: 15–30 μm (Kowalski and Butin 1989); *S.cracoviensis*: 26.8–63.9 μm) and shorter and wider ascospores (*S.prolifera*: 3.2–3.8 × 1.8–2 μm (Kowalski and Butin 1989); *S.cracoviensis*: 2.8–5.1 × 1–1.6 μm). The conidia of *S.prolifera* are also smaller (*S.prolifera*: 4–5.8 × 1.6–2.2 μm (Kowalski and Butin 1989) compared to *S.cracoviensis* (2.8–8.7 × 1.1–2.7 μm).

*Sporothrixcracoviensis* was represented by four isolates collected from adult *Trypodendrondomesticum* beetles on *Fagussylvatica*. It corresponds to *Sporothrix* sp. 7 in the study of Jankowiak et al. (2019a).

### Sporothrix fraxini

Taxon classificationFungiOphiostomatalesOphiostomataceae

R. Jankowiak sp. nov.

9A56C94E-8715-581B-8F9D-8B1FDEB9966F

840463

Fig. 7

#### Etymology.

From Latin, referring to the genus name of the host (*Fraxinusexcelsior*).

#### Type.

Poland, Małopolskie Province, Zbylitowska Góra, from the gallery of *Hylesinusvarius* on *Fraxinusexcelsior*, May 2016, *R. Jankowiak* (O-F-258630 ***holotype***, culture ex-type CBS 147936).

#### Description.

Sexual and asexual structures produced on sterilized ash twigs and on surface of malt agar in Petri dishes. *Ascomata* abundant, superficially or partly embedded in the agar, single or in groups; *ascomatal base* black, globose, (89–)110–161(–216) μm diam., with brown hyphal hairs, 14 to 65 μm long and 1.1 to 2.1 μm wide at the base; *ascomatal necks* black, straight or curved, (222–)332–461(–526) μm long, diameter (10.1–)11.3–16(–20.4) μm at the apex and (26.2–)29.1–41.4(–53) μm at the base. *Ostiolar hyphae* present, pale brown, septate, straight or rather tortuous, tapering towards the apex or sporadically dichotomous branching at the tip, (8–)10–20(–24) in number (21.4–)31.1–52.1(–73.6) μm long, (0.4–)0.7–1.1(–1.4) μm at the apex and (1.4–)1.8–2.4–(3.1) μm at the base. *Asci* evanescent. *Ascospores* one-celled, allantoid in side view (2.7–)2.9–3.5(–4.4) × (0.9–)1–1.4(–1.8) μm, elliptical in front view (2.2–)2.9–3.8(–4.7) × (0.8–)1.2–1.6(–1.8) μm, sometimes with residual sheath up to 1 μm thick, accumulated in white-color mass at the tip of the neck. *Conidiophores* hyaline, micronematous, simple or branched, straight, simple or branched, bearing several conidiogenous cells, either borne on vegetative hyphae or on upright hyphae. *Conidiogenous cells* blastic, cylindrical terminal or intercalary, straight or curved, tapering towards the apex, swollen apical part forming conidia by sympodial proliferation on hardly visible denticles, (13.6–)14.6–47.7(–99.6) μm long, (0.9–)1.2–1.6(–1.9) μm wide at the base. Apical part (0.8–)1.7–5.1(–10.6) μm long and (0.8–)1.1–2(–3) μm wide. *Conidia* hyaline, unicellular, smooth, obovoid to ellipsoidal, ends slightly rounded or truncate, (2.6–)3.4–5(–6.8) × (0.8–)1.1–1.6(–2) μm, formed directly on denticles. *Culture characteristics*: Cultures showing optimum growth at 25 °C (1 mm/d) followed by at 30 °C (0.9 mm/d), white, flat, growing in a circular pattern with smooth margins, with sparse aerial mycelium, often fading around the edges.

**Figure 7.**
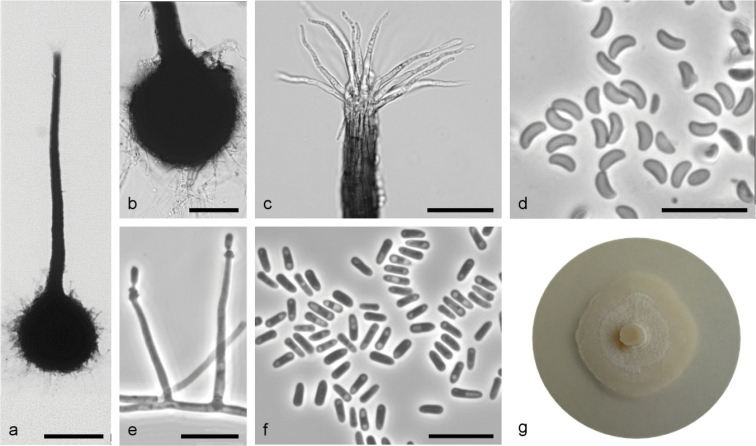
*Sporothrixfraxini* sp. nov. (CBS 147936) **a** ascoma **b** ascomatal base **c** ostiolar hyphae **d** ascospores **e** conidiogenous cell with an inflated cluster of denticles at the apex **f** conidia **g** fourteen-day-old culture on MEA. Scale bars: 100 μm (**a**), 50 μm (**b**), 25 μm (**c**), 10 μm (**d–f**).

#### Host tree.

*Fraxinusexcelsior*.

#### Insect vector.

*Hylesinuscrenatus*, *H.varius*.

#### Distribution.

Poland

#### Additional specimen examined.

Poland, Małopolskie Province, Zbylitowska Góra, from the gallery of *Hylesinusvarius* on *Fraxinusexcelsior*, May 2016, *R. Jankowiak* (O-F-258631, cultures CBS 147938).

#### Notes.

This species is phylogenetically distinct from the other *Sporothrix* species based on the ITS, βT, CAL and TEF1-α sequences. *Sporothrixfraxini* is closely related to *S.variecibatus*. However, *S.variecibatus* does not produce a sexual morph, and has narrower conidia (2–3 μm) (Roets et al. 2008) compared to *S.fraxini* (0.8–2 μm). In addition, the conidia of *S.variecibatus* are clavate while *S.fraxini* has obovoid to ellipsoidal conidia.

*Sporothrixfraxini* was represented by three isolates collected from the galleries of *Hylesinusvarius* on *Fraxinusexcelsior*. It corresponds to *Sporothrix* sp. 8 in the previous study of Jankowiak et al. (2019a).

### Sporothrix resoviensis

Taxon classificationFungiOphiostomatalesOphiostomataceae

R. Jankowiak & A. Ostafińska sp. nov.

FE138B30-1262-51AB-9F44-063D7FC05B43

840475

Fig. 8

#### Etymology.

From Latin, referring to the capital of Podkarpackie Voivodeship (Resovia in Latin, Rzeszów in Polish), the region from which this fungus was collected.

#### Type.

Poland, Podkarpackie Province, Borownica, from the wound on *Betulapendula*, June 2016, *A. Ostafińska*, (O-F-258632 ***holotype***, culture ex-type CBS 147927).

#### Description.

Sexual and asexual structures produced on sterilised birch twigs and on surface of malt agar in Petri dishes. *Ascomata* abundant, superficially or partly embedded in the agar, single or in groups; *ascomatal bases* black, globose, (87–)113–184(–232) μm diam., with brown hyphal hairs, 14 to 44 μm long and 0.9 to 2.2 μm wide at the base; *ascomatal necks* black, straight or curved, often extended at the base, (228–)378–624(–700) μm long, diameter (10–)11.2–17(–20.2) μm at the apex and (26.2–)34–47.7(–56) μm at the base. *Ostiolar hyphae* present, pale brown, septate, straight or curved, tapering towards the apex and often swollen at the tip, (7–)9–15(–18) in number, (15.7–)26.1–47.7(–67.6) μm long, (0.3–)0.7–1.5(–2.5) μm at the apex and (1.3–)2–3–(3.4) μm at the base. *Asci* evanescent. *Ascospores* one-celled, kidney-shaped to almost triangular in side view (2.7–)3.2–3.9(–4.4) × (1.4–)1.7–2.1(–2.3) μm, oblong-elliptical in front view (2.6–)3–3.8(–4.9) × (1.4–)1.7–2.2(–2.6) μm, without residual sheath accumulated in white-colored mass at the tip of the neck. *Conidiophores* hyaline, micronematous, straight, simple and bearing several conidiogenous cells, either borne on vegetative hyphae or on upright hyphae. *Conidiogenous cells* blastic, cylindrical, terminal, lateral or intercalary, straight or curved, swollen apical part forming conidia by sympodial proliferation on easily visible denticles, (3.1–)9.3–57(–120.1) μm long, (1–)1.1–1.6(–2.2) μm wide at the base. Apical part (1.3–)1.9–3.5(–4.4) μm long and (1.4–)2.4–3.9(–4.5) μm wide. *Conidia* hyaline, unicellular, smooth, obovate to ellipsoidal, pointed at the base, (3.9–)4.3–6.7(–8.5) × (2.1–)2.4–3.4(–4) μm, formed singly on denticles or on the side of vegetative hyphae. *Culture characteristics*: Cultures showing optimum growth at 25 °C (1.8 mm/d) followed by at 30 °C (1.7 mm/d), white, growing in a circular pattern with smooth margins, funiculose and woolly.

**Figure 8.**
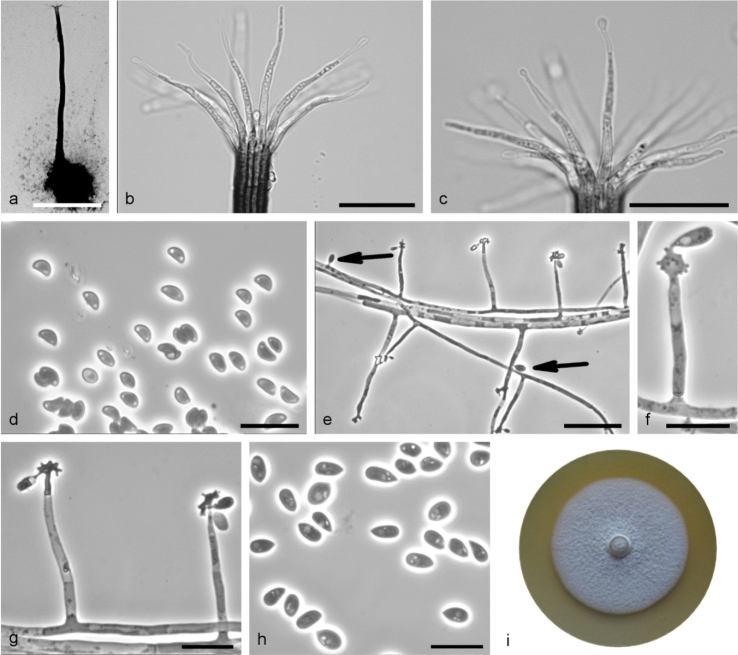
*Sporothrixresoviensis* sp. nov. (CBS 147927) **a** ascoma **b, c** ostiolar hyphae **d** ascospores **e–g** conidiogenous cell with an inflated cluster of denticles at the apex **h** conidia **i** fourteen-day-old culture on MEA. Scale bars: 250 μm (**a**), 25 μm (**b, c**), 10 μm (**d**), 25 μm (**e**), 10 μm (**f–h**).

#### Host trees.

*Betulapendula*.

#### Insect vector.

unknown.

#### Distribution.

Poland.

#### Note.

*Sporothrixresoviensis* is phylogenetically distinct from the other *Sporothrix* species based on the ITS, βT, CAL and TEF1-α sequences. This species grouped most closely with *S.stenoceras* but can be distinguished by its larger ascospores (*S.resoviensis*: 2.7–4.4 × 1.4–3.3 μm; *S.stenoceras*: 2.0–2.9 × 1.3–1.4 μm (Robak 1932). Perithecia developing on the agar medium and twigs have significantly shorter necks (*S.resoviensis*: 228–700 μm; *S.stenoceras*: 450–1500 μm (Robak 1932). *Sporothrixresoviensis* has larger conidia (3.9–8.5 × 2.1–4 μm) compared to *S.stenoceras* (3.4–6.9 × 2–3.4 μm). This new species also differs from *S.stenoceras* based on culture morphology, where *S.resoviensis* produces wooly cultures, different to the sparse and flat mycelium of *S.stenoceras* (Robak 1932).

*Sporothrixresoviensis* was represented by one isolate collected from a wound on *Betulapendula*. It corresponds to *Sporothrix* sp. 10 in the study of Jankowiak et al. (2019b).

### Sporothrix cryptarchum

Taxon classificationFungiOphiostomatalesOphiostomataceae

R. Jankowiak & A. Ostafińska sp. nov.

DA830A6A-94B4-55E9-BF76-8B7D1DE2C68D

840477

Fig. 9

#### Etymology.

Referring to the genus name of the beetle, *Cryptarcha* sp. (*Coleoptera*: *Nitidulidae*), with which this fungus is associated.

#### Type.

Poland, Małopolskie Province, Wierzchosławice, from *Cryptarchaundata* on *Quercusrobur*, June 2016, *R. Jankowiak*, (O-F-258633 ***holotype***, culture ex-type CBS 147934).

#### Description.

Sexual and asexual structures produced on the sterilised oak twigs and on the surface of malt agar in Petri dishes. *Ascomata* abundant, superficially or partly embedded in the agar, single or in groups; *ascomatal bases* black, globose, (55–)115–172(–210) μm diam., with brown hyphal hairs, 15 to 141 μm long and 0.9 to 3.8 μm wide at the base; *ascomatal necks* black, straight or curved, (126–)198–412(–544) μm long, diameter (10.9–)13–19(–23.8) μm at the apex and (17.6–)29.3–47.6(–59.6) μm at the base. *Ostiolar hyphae* present, pale brown, with small granules, septate, straight or curved, simple or dichotomous branching, tips tapering or sometimes thickened, (9–)13–24(–31) in number, (15.8–)30.5–51.8(–60.9) μm long, (0.2–)0.3–0.5(–0.7) μm at the apex and (0.9–)1.6–2.4–(3) μm at the base. *Asci* subglobose to ovoid, (5.5–)6.7–9(–11) × (4–)4.9–6.2(–7.2) μm. *Ascospores* one-celled, kidney-shaped to almost triangular in side view in side view (3.2–)3.8–4.7(–5.8) × (0.8–)1–1.3(–1.5) μm, elliptical in front view (3.1–)3.6–4.4(–5) × (1–)1.2–1.6(–1.8) μm, sometimes with residual sheath up to 0.6 μm thick, accumulated in white-colored mass at the tip of the neck. *Conidiophores* hyaline, micronematous, simple or occasionally branched and bearing several conidiogenous cells, either borne on vegetative hyphae or on upright hyphae. *Conidiogenous cells* blastic, cylindrical, terminal, lateral or intercalary, straight or curved, tapering towards the apex, swollen apical part forming conidia by sympodial proliferation on narrow denticles, (2.2–)13.9–51.2(–102.8) μm long, (0.7–)1.2–1.8(–2.2) μm wide at the base. Apical part (0.6–)1.4–3.1(–5.3) μm long and (1–)1.7–3(–3.8) μm wide, single denticles often below. Conidia of two types: 1) abundant in cultures, often produced, hyaline, unicellular, smooth, obovate to ellipsoid, pointed at the base, (3.3–)4.6–8.1(–10.3) × (1–)1.3–1.9(–2.2) μm, formed directly on denticles; 2) sparse in cultures, subhyaline to lightly pigmented, unicellular, smooth, subglobose to globose, (2.3–)3.1–4.1(–4.5) μm diam, formed singly, either directly on the side of vegetative hyphae or on short lateral branches. *Culture characteristics*: Cultures showing optimum growth at 25 °C (1.3 mm/d) followed by at 30 °C (1.1 mm/d), mostly pigmented or white or pig, flat, growing in a circular pattern with smooth margins.

**Figure 9.**
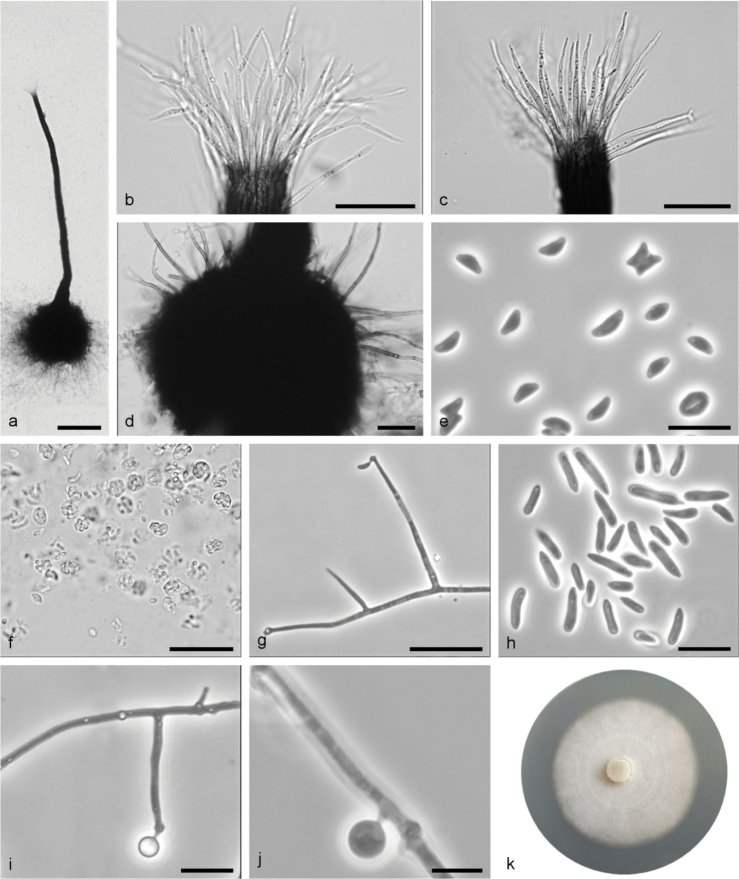
*Sporothrixcryptarchum* sp. nov. (CBS 147934) **a** ascoma **b** ascomatal base **c, d** ostiolar hyphae **e** ascospores **f** asci **g** conidiogenous cell with an inflated cluster of denticles at the apex **h** conidia **i** globose conidia arising on long conidiophore **j** globose conidia arising directly from hyphae **k** fourteen-day-old culture on MEA. Scale bars: 100 μm (**a**), 25 μm (**b–d**), 10 μm (**e**), 25 μm (**f, g**), 10 μm (**h, i**), 5 μm (**j**).

#### Host tree.

*Alnusglutinosa*, *Quercusrobur*.

#### Insect vector.

*Cryptarchaundata*, *C.strigata*.

#### Distribution.

Poland.

#### Additional specimen examined.

Poland, Małopolskie Province, Wierzchosławice, from *Cryptarchaundata* on *Quercusrobur*, June 2016, *R. Jankowiak*, (O-F-258634, cultures CBS 147933).

#### Notes.

This species is phylogenetically distinct from the other *Sporothrix* species based on the ITS, βT, CAL and TEF1-α sequences. *Sporothrixcryptarchum* is phylogenetically closely related to *S.undulata* (*Sporothrix* sp. 12) described in the present study. This species also shares morphological similarities such as kidney-shaped ascospores and two morphological forms of conidia with *S.undulata*. However, *S.cryptarchum* has narrow ascospores (0.8–1.5 µm) compared to *S.undulata* (1.1–2 µm). It also has distinct ostiolar hyphae, with those in *S.cryptarchum* often dichotomously branching while in *S.undulata* these hyphae occur only sporadically and do not have dichotomous branching. Both species produce hyaline and pigmented conidia. However, *S.cryptarchum* cultures are predominantly hyaline whereas those in pure cultures of *S.undulata* are mostly pigmented. Their conidial shapes in these two species are similar but their dimensions are distinct. *Sporothrixcryptarchum* has conidia that are smaller than those of *S.undulata*. In addition, cultures of *S.cryptarchum* are white and grow in a circular pattern with smooth margins while those of *S.undulata* grow in a circular pattern with undulate margins and some have grey pigmentation.

*Sporothrixcryptarchum* was represented by four isolates collected from Poland. It corresponds to *Sporothrix* sp. 11 in the study of Jankowiak et al. (2019b). *Sporothrixcryptarchum* was isolated from wounds on hardwood trees and nitidulid beetles (*Coleoptera*: *Nitidulidae*), which visited fresh wounds on *Quercusrobur*.

### Sporothrix undulata

Taxon classificationFungiOphiostomatalesOphiostomataceae

R. Jankowiak & A. Ostafińska sp. nov.

3BC030F9-9C2F-5B67-AF8C-CBF7E15B88FE

840478

Fig. 10

#### Etymology.

Referring to the aerial mycelium growing in undulating concentric zones on MEA.

#### Type.

Poland, Małopolskie Province, Wierzchosławice, from *Epuraeaguttata* on *Quercusrobur*, June 2016, *R. Jankowiak*, (O-F-258635 ***holotype***, culture ex-type CBS 147929).

#### Description.

Sexual and asexual structures produced on sterilised oak twigs and on surface of malt agar in Petri dishes. *Ascomata* abundant, superficially or partly embedded in the agar, single or in groups; *ascomatal base* black, globose, (65–)95–186(–223) μm diam., with brown hyphal hairs, 8 to 134 μm long and 1.2 to 3.1 μm wide at the base; *ascomatal necks* black, straight or curved, (114–)174–482(–697) μm long, diameter (9.1–)12.3–18.7(–24.2) μm at the apex and (14.7–)22–40.3(–58.7) μm at the base. *Ostiolar hyphae* present, pale brown, with small granules, septate, straight or slightly waved, tapering towards the apex or sporadically dichotomously branched at the tip, (9–)16–28(–31) in number, (29.4–)39.9–59.5(–72) μm long, (0.4–)0.6–1(–1.1) μm at the apex and (1.5–)1.8–2.7–(3.3) μm at the base. *Asci* subglobose to ovoid, (5.7–)6.7–8.5(–9.4) × (3.4–)4.4–5.8(–6.4) μm. *Ascospores* one-celled, kidney-shaped to almost triangular in side view (3.4–)3.8–4.6(–4.9) × (1.1–)1.4–1.7(–2) μm, elliptical in front view (3.2–)3.5–4.5(–5.6) × (0.9–)1.5–2.1(–2.8) μm, sometimes with residual sheath up to 0.6 μm thick, accumulated in white-colored mass at the tip of the neck. *Conidiophores* hyaline, micronematous or semimacronematous, simple or occasionally branched and bearing several conidiogenous cells, either borne on vegetative hyphae or on upright hyphae. *Conidiogenous cells* blastic, cylindrical, terminal, lateral or intercalary, straight or curved, slightly tapering towards the apex, swollen apical part forming conidia by sympodial proliferation on small or hardly visible denticles, (5.2–)11.3–50.4(–112.2) μm long, (0.9–)1.3–1.8(–2.1) μm wide at the base. Apical part (1.1–)1.6–3.4(–5.9) μm long and (1.1–)1.7–3.5(–5.4) μm wide. *Conidia* of two types: 1) sparsely in cultures, hyaline, unicellular, smooth, ellipsoid, pointed at the base, (3.2–)4.2–7.8(–11.7) × (1.4–)1.7–2.4(–3.5) μm, formed directly on denticles; 2) abundant in cultures, subhyaline to lightly pigmented, unicellular, smooth, subglobose to globose, sometimes pointed at the base, (2.1–)2.9–4.2(–5.5) μm diam, formed singly or in chains, either directly on the side of vegetative hyphae, on short lateral branches or denticles. *Culture characteristics*: Cultures showing optimum growth at 25 °C (1.2 mm/d) with growth somewhat slower at 20 °C and 30 °C (0.9 mm/d), white or white grey, flat, growing in a circular pattern with undulate margins.

**Figure 10.**
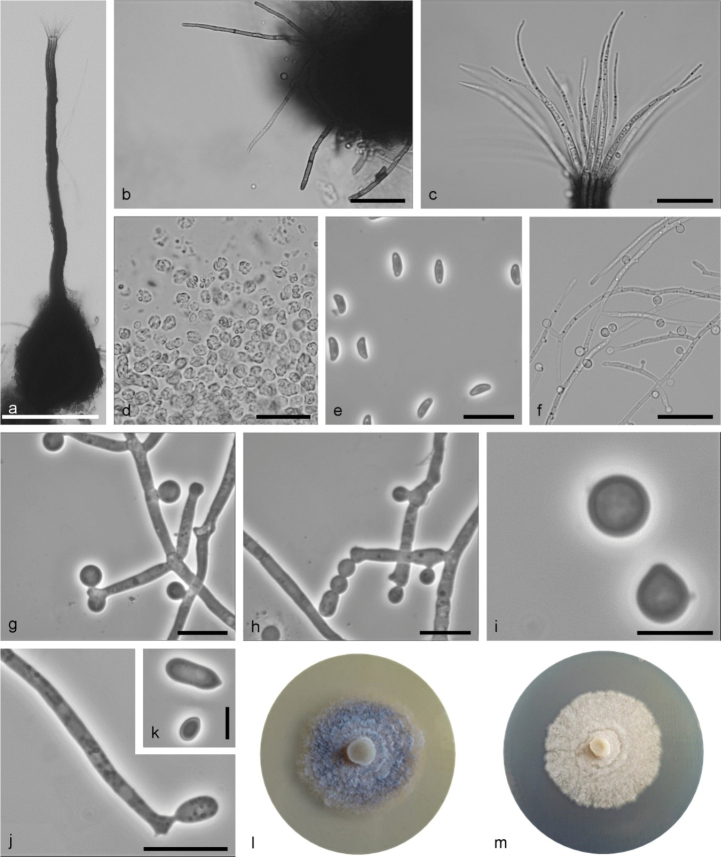
*Sporothrixundulata* sp. nov. (CBS 147929) **a** ascoma **b** ascomatal base **c** ostiolar hyphae **d** asci **e** ascospores **f–h** globose conidia arising on long conidiophore or directly from hyphae **i** globose conidia **j** conidiogenous cell with an inflated cluster of denticles at the apex **k** conidia **l–m** fourteen-day-old culture on MEA (left- pigmented CBS 147929, right – white KFL404DB16bRJCU). Scale bars: 100 μm (**a**), 25 μm (**b–d**), 10 μm (**e**), 25 μm (**f**), 10 μm (**g, h**), 5 μm (**i**), 10 μm (**j**), 5 μm (**k**).

#### Host tree.

*Alnusglutinosa*, *Carpinusbetulus*, *Fagussylvatica*, *Quercusrobur*, *Quercusrubra*, *Salixfragilis*.

#### Insect vector.

*Cryptarchaundata*, *Epuraeaguttata*.

#### Distribution.

Poland.

#### Additional specimen examined.

Poland, Małopolskie Province, Wierzchosławice, from wound on *Quercusrobur*, October 2015, *R. Jankowiak* (O-F-258636, cultures CBS 147931).

#### Notes.

This species is phylogenetically distinct from the other *Sporothrix* species based on the ITS, βT, CAL and TEF1-α sequences. *Sporothrixundulata* is phylogenetically closely related to *S.cryptarchum* described in this study. The morphological differences between *S.undulata* and *S.cryptarchum* are described in the section above treating *S.cryptarchum*.

*Sporothrixundulata* was represented by nine isolates collected from Poland. It corresponds to *Sporothrix* sp. 12 in the study of Jankowiak et al. (2019b). In this study *S.undulata* was isolated from wounds on hardwood trees and from adults of nitidulid beetles (*Coleoptera*: *Nitidulidae*), which visited wounds on *Quercusrobur*.

### Sporothrix cavum

Taxon classificationFungiOphiostomatalesOphiostomataceae

R. Jankowiak sp. nov.

BF96F230-1FED-5278-ACC6-99C26046F595

840479

Fig. 11

#### Etymology.

From Latin, referring to the hollow cavities produced by woodpeckers and from which this fungus was collected.

#### Type.

Poland, Małopolskie Province, Kraków, from the cavity of *Dendrocoposmajor* on *Salixfragilis*, December 2015, *R. Jankowiak*, (O-F-258637 ***holotype***, culture ex-type CBS 147943).

#### Description.

Sexual morph not observed. Asexual structures produced on sterilized beech twigs placed on the surface of malt agar in Petri dishes. *Conidiophores* hyaline, micronematous, simple, straight, simple or branched, bearing several conidiogenous cells, either borne on vegetative hyphae or on upright hyphae. *Conidiogenous cells* blastic, cylindrical, terminal, lateral or intercalary, straight or curved, slightly tapering toward the apex, swollen apical part forming conidia by sympodial proliferation on well-developed denticles, (2.8–)11.5–32.8(–54.4) μm long, (0.7–)1.1–1.7(–2.4) μm wide at the base. Apical part with denticles (1.2–)1.5–2.8(–4.4) μm long and (1.4–)1.8–2.6(–3.1) μm wide, individual denticles often formed below aplical part. *Conidia* hyaline, unicellular, smooth, obovoid, with pointed bases, (3.1–)3.6–5.5(–7.8) × (1.7–)2–2.7(–3.2) μm, formed on terminal or lateral denticles, either directly on the side of vegetative hyphae. *Culture characteristics*: Cultures having optimum growth at 25 °C (1.7 mm/d) followed by at 30 °C (1.5 mm/d), growing well at 35 °C (0.6 mm/d), greyish green, with a darker centre, flat, growing in a circular pattern with smooth margins and abundant aerial mycelium.

**Figure 11.**
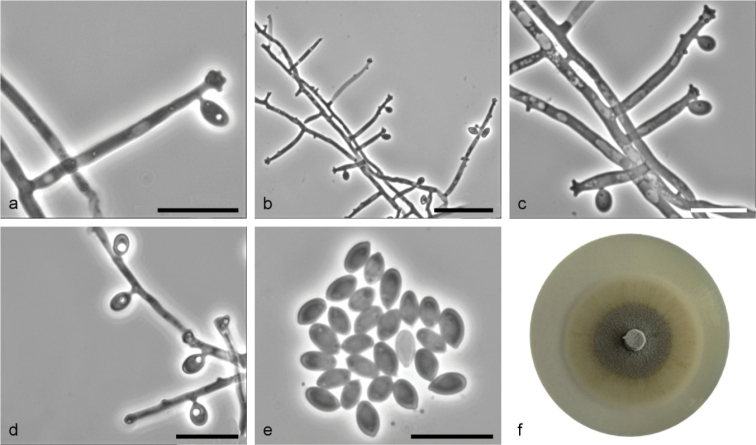
*Sporothrixcavum* sp. nov. (CBS 147943) **a–c** conidiogenous cell with an inflated cluster of denticles at the apex and below apex **d** conidiogenous cells arising directly from hyphae **e** conidia **f** fourteen-day-old culture on MEA. Scale bars: 10 μm (**a**), 25 μm (**b**), 10 μm (**c–e**).

#### Host tree.

*Malusdomestica*, *Salixfragilis*

#### Insect vector.

unknown

#### Distribution.

Poland

#### Additional specimen examined.

Poland, Małopolskie Province, Książ Wielki, from the cavity of *Dendrocoposmedius* on *Malusdomestica*, (O-F-258638, cultures ex-paratype KFL=NRFI 35614DR).

#### Notes.

This species is phylogenetically distinct from the other *Sporothrix* species based sequences for the ITS, βT, CAL and TEF1-α regions. *Sporothrixcavum* is related to *S.polyporicola* based on analyses of the ITS sequences. However, *S.cavum* in contrast to *S.polyporicola*, does not produce a sexual morph (Constantinescu and Ryman 1989). In addition, *S.cavum* has obovoid and short conidia (3.1–7.8 μm), whereas *S.polyporicola* has clavate and longer conidia (6–14 μm) (Constantinescu and Ryman 1989).

*Sporothrixcavum* was represented by two isolates collected from the cavities produced by the woodpeckers *Dendrocoposmajor* on *Salixfragilis* and *Dendrocoposmedius* on *Malusdomestica*. It corresponds to *Sporothrix* sp. 18 in the study of Jankowiak et al. (2019c).

## Discussion

Our work (Jankowiak et al. 2019a, 2019b, 2019c; this study) has led to the discovery of six novel *Sporothrix* species associated with hardwood trees in Poland. Description of these new species brings the total number of species in this genus to 62, of which 16 occur in Poland. These include the six species described here as well as *S.aurorae* (Jankowiak et al. 2019b), *S.cantabriensis* (Jankowiak et al. 2017), *S.dentifunda* (Aghayeva et al. 2005, Jankowiak et al. 2019b), *S.eucastaneae* (Jankowiak et al. 2019a, 2019b, 2021), *S.fusiformis* (Jankowiak et al. 2019a, 2019b), *S.inflata* (Jankowiak et al. 2012; Jankowiak and Bilański 2013a, 2013b), *S.inflata* ‘2’ (Jankowiak et al. 2019a, 2019b), *S.prolifera* (Kowalski and Butin 1989; Jankowiak et al. 2019a, 2019b), *S.stenoceras*, (Kowalski and Butin 1989; Jankowiak and Bilański 2013b, Jankowiak et al. 2019b) and *S.variecibatus* (Jankowiak and Bilański 2013b).

All of the species described in this study are morphologically similar, having asexual states with hyaline or lightly pigmented conidia produced holoblastically on denticulate conidiogenous cells or directly from the hyphae. Where ascomata were present, these tended to have globose bases with elongated necks terminating in long ostiolar hyphae and allantoid or kidney-shaped ascospores not surrounded by hyaline sheaths. All of the newly described species grew optimally at 25 °C and all also grew well at 30 °C on MEA. *Sporothrixundulata* and *S.cavum* differed from the other four species in having pigmented as opposed to white cultures on MEA. All of the newly described species were recovered from hardwood ecosystems in Poland in association with bark and ambrosia beetles, nitidulid beetles, naturally occurring tree wounds or woodpecker cavities.

The six species described in this study can easily be distinguished from each other and from the other species of *Sporothrix* based on the DNA sequence comparisons. Analyses of the ITS sequence data were insufficient to distinguish between *S.cryptarchum* and *S.undulata* or between *S.cracoviensis* and *S.fusiformis*. However, analyses of sequence data for the protein-coding genes, including the βT, CAL and TEF1-α showed that *S.cracoviensis*, *S.cryptarchum*, and *S.undulata* represent distinct taxa. Furthermore, the two closely related species, *S.cryptarchum* and *S.undulata* formed a new and well-supported lineage in *Sporothrix* including species infecting wounds on a variety of hardwood trees. The species in this lineage are characterised by having both hyaline as well as pigmented conidia and kidney-shaped ascospores.

The asexual morphs of the *Sporothrix* species described in this study had variable morphology. All species had hyaline conidia produced holoblastically on denticulate conidiogenous cells that proliferate sympodially or arise directly from hyphae. *Sporothrixcryptarchum* and *S.undulata* also had pigmented globose conidia formed singly or in chains, either directly on the sides of the vegetative hyphae or on short lateral branches. The presence of two different conidial types has previously been found in other *Sporothrix* species, including *Sporothrixdimorphospora* and *S.brunneoviolacea* (Madrid et al. 2010) as well as *S.brasiliensis*, *S.globose*, and *S.mexicana* (Marimon et al. 2007).

Recently, Jankowiak et al. (2019b) provided evidence that fresh wounds on hardwood trees in Europe are preferred habitats for some *Sporothrix* species. These authors isolated 15 *Sporothrix* species from trees belonging to 12 species of angiosperms. Likewise, nine *Sporothrix* species have been described from fresh wounds on non-native *Eucalyptus* spp. and various genera of native trees in South Africa (Kamgan Nkuekam et al. 2012; Musvuugwa et al. 2016, 2020; Osorio et al. 2016).

Three species of wound-associated *Sporothrix* spp. collected during a survey reported in the study of Jankowiak et al. (2019b) were included in the present study. The greatest number of isolates (194) obtained during that survey were those of *S.undulata*. This species was found as a common associate of bleeding wounds on *Quercusrobur* and *Salixfragilis*, suggesting that they might have some level of pathogenicity. The other species inhabiting wounds on hardwood trees that was collected during the survey of Jankowiak et al. (2019a) was *S.cryptarchum* (34 isolates). Transfer of this species to the sampled tree wounds was most likely by nitidulid (*Coleoptera*, *Nitidulidae*) beetles as previously noted by Jankowiak et al. (2019b) who suggested that these insects commonly transmit *Ophiostomatales*, including *Sporothrix* species to tree wounds in Poland. Likewise, Kamgan Nkuekam et al. (2012) have demonstrated that the nitidulid beetles *Brachypeplusdepressus* and *Carpophilus* spp. vector *S.candida* and *S.fumea* in the *Eucalyptus* plantations of South Africa. This association is also consistent with other studies providing compelling evidence that nitidulid beetles act as vectors of the well-known pathogens, such as *Bretziellafagacearum* (De Beer et al. 2017; Jagemann et al. 2018) and *Ceratocystisalbifundus* (Heath et al. 2009).

The second largest number of isolates (81 in total) included in this study represented two species in the *S.gossypina*-complex, bringing the total number of species in that complex to 15 (De Beer et al. 2016; Wang et al. 2019). *Sporothrixcracoviensis* was represented by 45 isolates from the ambrosia beetles *Trypodendrondomesticum* and *T.signatum* collected on *Fagussylvatica* (Jankowiak et al. 2019a). This is not unusual given that an association between ambrosia beetles has recently been recorded by De Errasti et al.(2016) in a study on *Nothofaguspumelo* in Patagonia. The other species residing in this complex collected during the survey of Jankowiak et al. (2019a) is *S.fraxini* (36 isolates). This fungus was found on *Fraxinusexcelsior* in association with the bark beetles *Hylesinuscrenatus* and *H.varius* (Jankowiak et. al. 2019a).

The Polish study by Jankowiak et al. (2019a) revealed that, apart from *S.cracoviensis* and *S.fraxini*, five other *Sporothrix* species (*S.fusiformis*, *S.prolifera*, *S.eucastanea*, *Sporothrix* sp. 4, *Sporothrix* sp. 9) were associated with bark beetles. These findings confirm that most species in the *S.gossypina* complex are associated with galleries of conifer-infesting bark beetles worldwide (De Beer et al. 2016). The other species in the *S.gossypina*-complex were isolated from the stained oak wood (Kowalski and Butin 1989; Aghayeva et al. 2004), cankers caused by *Cryphonectriaparasitica* on chestnut (Davidson 1978), a hardwood tree native to South Africa (Musvuugwa et al. 2016), and from mites infesting the infructescences (flower heads) of *Protea* in South Africa (Roets et al. 2008).

*Sporothrixcavum*, the remaining taxon collected from hardwood trees during the surveys that formed the basis of the present study, resided in lineage F defined by De Beer et al. (2016). This lineage includes three species, namely *S.polyporicola*, *S.dimorphospora*, and *S.inflata* ‘2’. Two of these species (*S.dimorphospora*, and *S.inflata* ‘2’) are known from soil and *S.polyporicola* was isolated from basidiocarps of the polypores *Fomitopsispinicola* and *Amaropostiastiptica* (Constantinescu and Ryman 1989; Madrid et al. 2010). The results of the present study show that species in this complex also accommodate wood-inhabiting *Sporothrix* species. Other than the fact that *S.cavum* was isolated from cavities on *Salixfragilis* and *Malusdomestica* made by woodpeckers (Jankowiak et al. 2019c), nothing is known regarding the ecology or distribution of the fungus. It could, for example, be introduced into these cavities by arthropods or have some relationship with the woodpeckers themselves.

The results of this study have substantially expanded our knowledge of *Sporothrix* and the ecology of species in this genus. Broadly, the results suggest that *Sporothrix* species are common members of the *Ophiostomatales* in hardwood ecosystems in Poland. Furthermore, interesting questions have arisen that should shape future investigations regarding these fungi.

## Supplementary Material

XML Treatment for Sporothrix cracoviensis

XML Treatment for Sporothrix fraxini

XML Treatment for Sporothrix resoviensis

XML Treatment for Sporothrix cryptarchum

XML Treatment for Sporothrix undulata

XML Treatment for Sporothrix cavum

## References

[B1] Aghayeva DN Wingfield MJ De Beer WZ Kirisits T (2004) Two new *Ophiostoma* species with *Sporothrix* anamorphs from Austria and Azerbaijan. Mycologia 96: 866–878. 10.1080/15572536.2005.11832933 21148906

[B2] Aghayeva DN Wingfield MJ Kirisits T Wingfield BD (2005) *Ophiostomadentifundum* sp. nov. from oak in Europe, characterized using molecular phylogenetic data and morphology. Mycological Research 109: 1127–1136. 10.1017/S0953756205003710 16279407

[B3] Bakshi BK (1950) Fungi associated with Ambrosia beetles in Great Britain. Transactions of the British Mycological Society 33: 111–120. 10.1016/S0007-1536(50)80054-2

[B4] Barros MBdL Schubach AdO do Valle ACF Gutierrez Galhardo MC Conceição-Silva F Schubach TM Reis RS Wanke B Marzochi KB Conceição MJ (2004) Cat-transmitted sporotrichosis epidemic in Rio de Janeiro, Brazil: description of a series of cases. Clinical Infectious Diseases 38: 529–535. 10.1086/381200 14765346

[B5] Berbee ML Taylor JW (1992) 18s Ribosomal RNA gene sequence characters place the human pathogen *Sporothrixschenckii* in the genus *Ophiostoma*. Experimental Mycology 16: 87–91. 10.1016/0147-5975(92)90044-R

[B6] Carbone I Kohn LM (1999) A method for designing primer sets for speciation studies in filamentous ascomycetes. Mycologia 91: 553–556. 10.2307/3761358

[B7] Constantinescu O Ryman S (1989) A new *Ophiostoma* on polypores. Mycotaxon 34: 637–642.

[B8] Darriba D Taboada GL Doallo R Posada D (2012) jModelTest 2: more models, new heuristics and parallel computing. Nature Methods 9: 772–772. 10.1038/nmeth.2109 PMC4594756 22847109

[B9] Davidson RW (1942) Some additional species of *Ceratostomella* in the United States. Mycologia 34: 650–662. 10.1080/00275514.1942.12020934

[B10] Davidson RW (1978) A new species of *Ceratocystis* on *Endothiaparasitica* canker of American chestnut. Mycologia 70: 856–858. 10.2307/3759367

[B11] De Beer ZW Harrington TC Vismer HF Vingfield BD Wingfield MJ (2003) Phylogeny of the *Ophiostomastenoceras*–*Sporothrixschenckii* complex. Mycologia 95: 434–441. 10.2307/3761885 21156632

[B12] De Beer ZW Wingfield MJ (2013) Emerging lineages in the Ophiostomatales. In: Seifert KA De Beer ZW Wingfield MJ (Eds) The Ophiostomatoid Fungi: Expanding Frontiers.Centraalbureau voor Schimmelcultures (CBS), Utrecht, the Netherlands: CBS Biodiversity Series: 12: 21–46.

[B13] De Beer ZW Seifert KA Wingfield MJ (2013a) The ophiostomatoid fungi: their dual position in the Sordariomycetes. In: Seifert KA De Beer ZW Wingfield MJ (Eds) Ophiostomatoid fungi: Expanding Frontiers.CBS Biodiversity Series 12: 1–19.

[B14] De Beer ZW Seifert KA Wingfield MJ (2013b) A nomenclator for ophiostomatoid genera and species in the Ophiostomatales and Microascales. In: Seifert KA De Beer ZW Wingfield MJ (Eds) The Ophiostomatoid Fungi: Expanding Frontiers.Centraalbureau voor Schimmelcultures (CBS), Utrecht, the Netherlands, CBS Biodiversity Series 12: 245–322.

[B15] De Beer ZW Duong TA Wingfield MJ (2016) The divorce of *Sporothrix* and *Ophiostoma*: solution to a problematic relationship. Studies in Mycology 83: 165–191. 10.1016/j.simyco.2016.07.001 27616802 PMC5007658

[B16] De Beer ZW Marincowitz S Duong TA Wingfield MJ (2017) *Bretziella*, a new genus to accommodate the oak wilt fungus, *Ceratocystisfagacearum* (Microascales, Ascomycota). MycoKeys 27: 1–19. 10.3897/mycokeys.27.20657

[B17] De Beurmann L Gougerot H (1911) Les Sporotrichum pathogènes. Classification botanique. Archives de Parasitologie 15: 5–109.

[B18] De Errasti A De Beer ZW Coetzee MPA Roux J Rajchenberg M Wingfield MJ (2016) Three new species of Ophiostomatales from *Nothofagus* in Patagonia. Mycological Progress 15: e17. 10.1007/s11557-016-1158-z

[B19] de Hoog GS (1974) The genera *Blastobotrys*, *Sporothrix*, *Calcarisporium* and *Calcarisporiella* gen. nov. Studies in Mycology 7: 1–84.

[B20] de Hoog GS Rantio-Lehtimäki AH Smith MT (1985) *Blastobotrys*, *Sporothrix* and *Trichosporiella*: generic delimitation, new species, and a *Stephanoascus* teleomorph. Antonie van Leeuwenhoek 51: 79–109. 10.1007/BF00444231 4039915

[B21] De Meyer EM De Beer ZW Summerbell RC Moharram AM de Hoog GS Vismer HF Wingfield MJ (2008) Taxonomy and phylogeny of new wood- and soil-inhabiting *Sporothrix* species in the *Ophiostomastenoceras*–*Sporothrixschenckii* complex. Mycologia 100: 647–661. 10.3852/07-157R 18833758

[B22] Gardes M Bruns TD (1993) ITS primers with enhanced speciﬁcity for basidiomycetes: application to the identiﬁcation of mycorrhizae and rusts. Molecular Ecology 2: 113–118. 10.1111/j.1365-294X.1993.tb00005.x 8180733

[B23] Georgescu CC Teodoru I Badea M (1948) Uscarea in massa a stejarului. Ciupere de alteratie cromatica parazitara a lemnului de stejar. An alele Institutului de Cercetari Forestiere al României 11: 185–217.

[B24] Glass NL Donaldson GC (1995) Development of primer sets designed for use with the PCR to amplify conserved genes from filamentous ascomycetes. Applied and Environmental Microbiology 61: 1323–1330. 10.1128/aem.61.4.1323-1330.1995 7747954 PMC167388

[B25] Goidànich G (1935) Una nuova specie di “Ophiostoma” vivente sul pero ed alcune osservazioni sull’esatta posizione istematica della forma ascofora e delle forme metagenetiche del genere. Bolletino della Stazione di Patología Vegetale di Roma 15: 122–168.

[B26] Grobbelaar JW Aghayeva DN De Beer ZW Bloomer P Wingfield MJ Wingfield BD (2009) Delimitation of *Ophiostomaquercus* and its synonyms using multiple gene phylogenies. Mycological Progress 8: 221–236. 10.1007/s11557-009-0594-4

[B27] Guindon S Dufayard JF Lefort V Anisimova M Hordijk W Gascuel O (2010) New algorithms and methods to estimate maximum-likelihood phylogenies: assessing the performance of PhyML 3.0. Systematic Biology 59: 307–321. 10.1093/sysbio/syq010 20525638

[B28] Guindon S Gascuel O (2003) A simple, fast and accurate method to estimate large phylogenies by maximum-likelihood. Systematic Biology 52: 696–704. 10.1080/10635150390235520 14530136

[B29] Hall TA (1999) BioEdit: a user-friendly biological sequence alignment editor and analysis program for Windows 95/98/NT. Nucleic Acids Symposium 41: 95–98. https://doi: 10.14601/Phytopathol_Mediterr-14998u1.29

[B30] Hausner G Reid J Klassen GR (1993) On the phylogeny of *Ophiostoma*, *Ceratocystis**s.s.*, and *Microascus*, and relationships within *Ophiostoma* based on partial ribosomal DNA sequences. Canadian Journal of Botany 71: 1249–1265. 10.1139/b93-148

[B31] Hausner G Reid J Klassen GR (2000) On the phylogeny of members of *Ceratocystis**s.s.* and *Ophiostoma* that possess different anamorphic states, with emphasis on the anamorph genes *Leptographium*, based on partial ribosomal DNA sequences. Canadian Journal of Botany 78: 903–916. 10.1139/b00-068

[B32] Heath RN Wingfiled MJ Van Wyk M Roux J (2009) Insect Associates of *Ceratocystisalbifundus* and Patterns of Association in a Native Savanna Ecosystem in South Africa. Environmental Entomology 38: 356–364. 10.1603/022.038.0207 19389283

[B33] Hedgcock GG (1906) Studies upon some chromogenic fungi which discolor wood. Missouri Botanical Garden Annual Report 17: 59–114. 10.2307/2400089

[B34] Hektoen L Perkins CF (1900) Refractory subcutaneous abscesses caused by *Sporothrixschenckii*, a new pathogenic fungus. Journal of Experimental Medicine 5: 77–89. 10.1084/jem.5.1.77 PMC2117997 19866937

[B35] Hunt J (1956) Taxonomy of the genus *Ceratocystis*. Lloydia 19: 1–58.

[B36] Jagemann SM Juzwik J Tobin PC Raffa KF (2018) Seasonal and regional distributions, degree-day models, and phoresy rates of the major sap beetle (Coleoptera: Nitidulidae) vectors of the oak wilt fungus, *Bretziellafagacearum*, in Wisconsin. Environmental Entomology 47: 1152–1164. 10.1093/ee/nvy080 29905833

[B37] Jankowiak R Bilański P Kolařik M Wasiuta D (2012) Root-colonizing ophiostomatoid fungi associated with dying and dead young Scots pine in Poland. Forest Pathology 42: 492–500. 10.1111/j.1439-0329.2012.00783.x

[B38] Jankowiak R Bilański P (2013a) Association of the pine-infesting *Pissodes* species with ophiostomatoid fungi in Poland. European Journal of Forest Research 132: 523–534. 10.1007/s10342-013-0693-2

[B39] Jankowiak R Bilański P (2013b) Diversity of ophiostomatoid fungi associated with the large pine weevil, *Hylobiusabietis*, and infested Scots pine seedlings in Poland. Annals of Forest Science 70: 391–402. 10.1007/s13595-013-0266-z

[B40] Jankowiak R Strzałka B Bilański P Kacprzyk M Lukášová K Linnakoski R Matwiejczuk S Misztela M Rossa R (2017) Diversity of Ophiostomatales species associated with conifer-infesting beetles in the Western Carpathians. European Journal of Forest Research 136: 939–956. 10.1007/s10342-017-1081-0

[B41] Jankowiak R Strzałka B Bilański P Kacprzyk M Wieczorek P Linnakoski R (2019a) Ophiostomatoid fungi associated with hardwood-infesting bark and ambrosia beetles in Poland: taxonomic diversity and vector specificity. Fungal Ecology 39: 152–167. 10.1016/j.funeco.2019.02.001

[B42] Jankowiak R Bilański P Ostafińska A Linnakoski R (2019b) Ophiostomatales associated with wounds on hardwood trees in Poland. Plant Pathology 68: 1407–1424. 10.1111/ppa.13061

[B43] Jankowiak R Ciach M Bilański P Linnakoski R (2019c) Diversity of wood-inhabiting fungi in woodpecker nest cavities in southern Poland. Acta Mycologica 54: e1126. 10.5586/am.1126

[B44] Jankowiak R Bilański P Strzałka B Linnakoski R Bosak A Hausner G (2019d) Four new *Ophiostoma* species associated with conifer- and hardwood-infesting bark and ambrosia beetles from Czech Republic and Poland. Antonie van Leeuwenhoek 112: 1501–15021. 10.1007/s10482-019-01277-5 31140027 PMC6748885

[B45] Jankowiak R Szewczyk G Bilański P Jazłowiecka D Harabin B Linnakoski R (2021) Blue-stain fungi isolated from freshly felled Scots pine logs in Poland, including *Leptographiumsosnaicola* sp. nov. Forest Pathology 51(2): e12672. 10.1111/efp.12672

[B46] Kamgan Nkuekam G De Beer ZW Wingfield MJ Mohammed C Carnegie AJ Pegg GS Roux J (2011) *Ophiostoma* species (Ophiostomatales, Ascomycota), including two new taxa on eucalypts in Australia. Australian Journal of Botany 59: 283–297. 10.1071/BT10231

[B47] Kamgan Nkuekam G De Beer ZW Wingfield MJ Roux J (2012) A diverse assemblage of *Ophiostoma* species, including two new taxa on eucalypt trees in South Africa. Mycological Progress 11: 515–533. 10.1007/s11557-011-0767-9

[B48] Katoh K Standley DM (2013) MAFFT multiple sequence alignment software version 7, improvements in performance and usability. Molecular Biology and Evolution 30: 772–780. 10.1093/molbev/mst010 23329690 PMC3603318

[B49] Kornerup A Wanscher JH (1978) Methuen Handbook of Colour. 3^rd^ edn. Eyre Methuen, London, 252 pp.

[B50] Kowalski T Butin H (1989) Taxonomie bekannter und neuer *Ceratocystis*-Arten an Eiche (*Quercusrobur* L.). Journal of Phytophathology 124: 236–248. 10.1111/j.1439-0434.1989.tb04919.x

[B51] Lagerberg T Lundberg G Melin E (1927) Biological and practical researches into blueing in pine and spruce. Svenska Skogsvårdsföreningens Tidskrift 25: 145–272.

[B52] Linnakoski R De Beer ZW Ahtiainen J Sidorov E Niemelä P Pappinen A Wingfield MJ (2010) *Ophiostoma* spp. associated with pine- and spruce-infesting bark beetles in Finland and Russia. Persoonia 25: 72–93. 10.3767/003158510X550845 21339968 PMC3028507

[B53] Lòpez-Romero E Reyes-Montes MdR Pèrez-Torres A Ruiz-Baca E Villagómez-Castro JC Mora-Montes HM Flores-Carreón A Toriello C (2011) *Sporothrixschenckii* complex and sporotrichosis, an emerging health problem. Future Microbiology 6: 85–102. 10.2217/fmb.10.157 21162638

[B54] Madrid H Gene J Cano J Silvera C Guarro J (2010) *Sporothrixbrunneoviolacea* and *Sporothrixdimorphospora*, two new members of the *Ophiostomastenoceras*–*Sporothrixschenckii* complex. Mycologia 102: 1193–1203. 10.3852/09-320 20943519

[B55] Marimon R Cano J Gene J Sutton DA Kawasaki M Guarro J (2007) *Sporothrixbrasiliensis*, *S.globosa*, and *S.mexicana*, three new *Sporothrix* species of clinical interest. Journal of Clinical Microbiology 45: 3198–3206. 10.1128/JCM.00808-07 17687013 PMC2045377

[B56] Marmolejo JG Butin H (1990) New conifer-inhabiting species of *Ophiostoma* and *Ceratocystiopsis* (Ascomycetes, Microascales) from Mexico. Sydowia 42: 193–199.

[B57] Mathiesen-Käärik A (1953) Eine Übersicht über die gewöhnlichsten mit Borkenkäfern assoziierten Bläuepilze in Schweden und einige für Schweden neue Bläuepilze. Meddelanden från Statens Skogsforskningsinstitut 43: 1–74.

[B58] Melin E Nannfeldt JA (1934) Researches into the blueing of ground wood-pulp. Svenska Skogsvårdsföreningens Tidskrift 32: 397–616.

[B59] Münch E (1907) Die Blaufäule des Nadelhozes. I–II. Naturwissenschaftliche Zeitschrift für Forst- und Landwirtschaft 5: 531–573.

[B60] Musvuugwa T De Beer ZW Duong TA Dreyer LL Oberlander K Roets F (2016) Wounds on *Rapaneamelanophloeos* provide habitat for a large diversity of ophiostomatales including four new species. Antonie van Leeuwenhoek 109: 877–894. 10.1007/s10482-016-0687-4 27022984

[B61] Musvuugwa T de Beer ZW Dreyer LL Duong T Marincowitz S Oberlander KC Roets F (2020) New ophiostomatoid fungi from wounds on storm-damaged trees in Afromontane forests of the Cape Floristic Region. Mycological Progress 19: 81–95. 10.1007/s11557-019-01545-8

[B62] Ngubane NP Dreyer LL Oberlander KC Roets F (2018) Two new *Sporothrix* species from *Protea* flower heads in South African Grassland and Savanna. Antonie van Leeuwenhoek 111: 965–979. 10.1007/s10482-017-0995-3 29214366

[B63] O’Donnell K Cigelnik E (1997) Two divergent intragenomic rDNA ITS2 types within a monophyletic lineage of the fungus *Fusarium* are nonorthologous. Molecular Phylogenetics and Evolution 7: 103–116. 10.1006/mpev.1996.0376 9007025

[B64] O’Donnell K Kistler HC Cigelnik E Ploetz RC (1998) Multiple evolutionary origins of the fungus causing Panama disease of banana: concordant evidence from nuclear and mitochondrial gene genealogies. Proceedings of the National Academy of Sciences of the United States of America 95: 2044–2049. 10.1073/pnas.95.5.2044 9482835 PMC19243

[B65] O’Donnell K Nirenberg H Aoki T Cigelnik E (2000) A multigene phylogeny of the *Gibberellafujikuroi* species complex: detection of additional phylogenetically distinct species. Mycoscience 41: 61–78. 10.1007/BF02464387

[B66] Osorio JA De Beer ZW Wingfield M Roux J (2016) Ophiostomatoid fungi associated with mangroves in South Africa, including *Ophiostomapalustre* sp. nov. Antonie van Leeuwenhoek 109: 1555–1571. 10.1007/s10482-016-0757-7 27562287

[B67] Rambaut A Drummond AJ (2007) Tracer v1.4. http://beast.bio.ed.ac.uk/Tracer

[B68] Robak H (1932) Investigations regarding fungi on Norwegian ground wood pulp and fungal infection at wood pulp mills. Nyt Magazin for Naturvidenskaberne 71: 185–330.

[B69] Roets F De Beer ZW Dreyer LL Zipfel R Crous PW Wingfield MJ (2006) Multi-gene phylogeny for *Ophiostoma* spp. reveals two new species from *Proteainfructescences*. Studies in Mycology 55: 199–212. 10.3114/sim.55.1.199 18490980 PMC2104725

[B70] Roets F De Beer ZW Wingfield MJ Crous PW Dreyer LL (2008) *Ophiostomagemellus* and *Sporothrixvariecibatus* from mites infesting *Proteainfructescences* in South Africa. Mycologia 100: 496–510. https://www.jstor.org/stable/20444973 18751556 10.3852/07-181r

[B71] Roets F Crous PW Wingfield MJ Dreyer LL (2009) Mite-mediated hyperphoretic dispersal of *Ophiostoma* spp. from the Infructescences of South African *Protea* spp. Environmental Entomology 38: 143–152. 10.1603/022.038.0118 19791608

[B72] Roets F Wingfield MJ Crous PW Dreyer LL (2013) Taxonomy and ecology of ophiostomatoid fungi associated with *Proteainfructescences*. In: Seifert KA, De Beer ZW, Wingfield MJ (Eds) The Ophiostomatoid Fungi: Expanding Frontiers Centraalbureau voor Schimmelcultures (CBS), Utrecht, the Netherlands, CBS Biodiversity Series 12: 177–187.

[B73] Ronquist F Huelsenbeck JP (2003) MrBayes 3: Bayesian phylogenetic inference under mixed models. Bioinformatics 19: 1572–1574. 10.1093/bioinformatics/btg180 12912839

[B74] Sczerbin-Parfenenko AL (1953) Rakovye i sosudistye bolezni listvennych porod. Goslesbumizdat, Moskva-Leningrad.

[B75] Siemaszko W (1939) Zespoły grzybów towarzyszących kornikom polskim. Planta Polonica 7: 1–54.

[B76] Summerbell RC Kane J Krajden S Duke EE (1993) Medically important *Sporothrix* species and related ophiostomatoid fungi. In: Wingfield MJ, Seifert KA, Webber J (Eds) *Ceratocystis* and *Ophiostoma*: taxonomy, ecology and pathogenicity. APS Press, St. Paul, 185–192.

[B77] Swofford DL (2003) PAUP* 4.0: phylogenetic analysis using parsimony (*and other methods). Sinauer Associates, Sunderland.

[B78] Travassos LR Lloyd KO (1980) *Sporothrixschenckii* and related species of *Ceratocystis*. Microbiological Reviews 44: 683–721. 10.1128/mr.44.4.683-721.1980 7010114 PMC373199

[B79] Villarreal M Rubio V De Troya MT Arenal F (2005) A new *Ophiostoma* species isolated from *Pinuspinaster* in the Iberian Peninsula. Mycotaxon 92: 259–268. http://hdl.handle.net/10261/18058

[B80] Wang HM Wang Z Liu F Wu CX Zhang SF Kong XB Decock C Lu Q Zhang Z (2019) Differential patterns of ophiostomatoid fungal communities associated with three sympatric *Tomicus* species infesting pines in south-western China, with a description of four new species. MycoKeys 50: 93–133. 10.3897/mycokeys.50.32653 31043857 PMC6477840

[B81] White TJ Bruns T Lee S Taylor J (1990) Amplification and direct sequencing of fungal ribosomal RNA genes for phylogenetics. In: Innis MA Gelfand DH Sninsky JJ White TJ (Eds) PCR protocols: a guide to methods and applications.Academic Press, San Diego, 315–322. 10.1016/B978-0-12-372180-8.50042-1

[B82] Zhang Y Hagen F Stielow B Rodrigues AM Samerpitak K Zhou X Feng P Yang L Chen M Deng S Li S Liao W Li R Li F Meis JF Guarro J Teixeira M Al-Zahrani HS Pires de Camargo Z Zhang L de Hoog GS (2015) Phylogeography and evolutionary patterns in *Sporothrix* spanning more than 14 000 human and animal case reports. Persoonia 35: 1–20. 10.3767/003158515X687416 26823625 PMC4713101

[B83] Zhou XD De Beer ZW Wingfield MJ (2006) DNA sequence comparisons of *Ophiostoma* spp., including *Ophiostomaaurorae* sp. nov., associated with pine bark beetles in South Africa. Studies in Mycology 55: 269–277. 10.3114/sim.55.1.269 18490985 PMC2104716

[B84] Zipfel RD De Beer ZW Jacobs K Wingfield BD Wingfield MJ (2006) Multi-gene phylogenies define *Ceratocystiopsis* and *Grosmannia* distinct from *Ophiostoma*. Studies in Mycology 55: 75–97. 10.3114/sim.55.1.75 18490973 PMC2104718

